# Electrochemical and Spectroscopic Characterization of Co-Neuroglobin: A Bioelectrocatalyst for H_2_ Production

**DOI:** 10.1021/acs.inorgchem.5c00551

**Published:** 2025-05-02

**Authors:** Mirco Meglioli, Federico Sebastiani, Marzia Bellei, Giulia Di Rocco, Antonio Ranieri, Carlo Augusto Bortolotti, Marco Sola, Marco Borsari, Giulietta Smulevich, Gianantonio Battistuzzi

**Affiliations:** † Department of Chemical and Geological Sciences, University of Modena and Reggio Emilia, via Campi 103, Modena 41125, Italy; ‡ Department of Chemistry “Ugo Schiff” DICUS, University of Florence, via della Lastruccia 3, Sesto Fiorentino (FI) 50019, Italy; § INSTM Research Unit of Firenze, via della Lastruccia 3, Sesto Fiorentino I-50019, Italy; ∥ Department of Life Sciences, University of Modena and Reggio Emilia, via Campi 103, Modena 41125, Italy

## Abstract

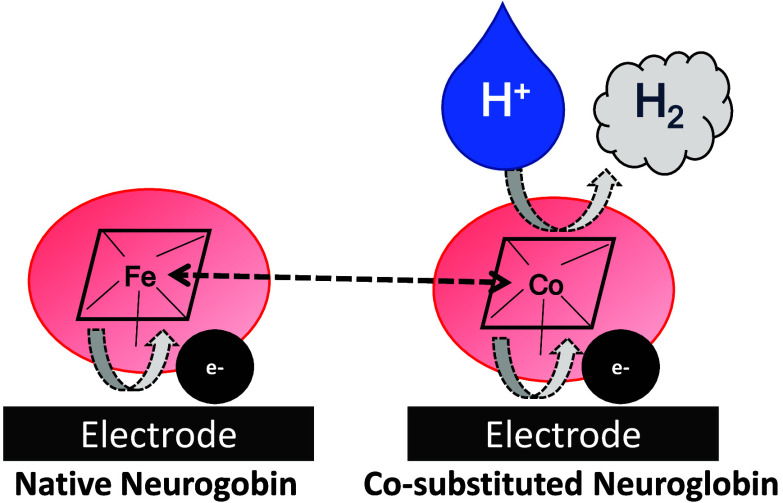

The electronic absorption, MCD, and RR spectra of the Co(III) and Co(II) derivatives of wild-type human neuroglobin (Co-WT) and its C46A/C55A mutant (Co–C46AC55A) were thoroughly investigated and compared with those of the corresponding Fe species and of the few Co-substituted heme proteins characterized so far. In both oxidation states, Co-WT and Co–C46AC55A contain a low-spin six-coordinated Co ion, whose axial coordination positions appear to be occupied by the distal and proximal histidines and whose electronic properties are scarcely affected by deletion of the C46–C55 disulfide bond. Both Co-WT and Co–C46AC55A feature negative *E*°′_Co(III)/Co(II)_ values. Fe(III) to Co(III) swapping does not significantly alter the pH dependence of their spectroscopic properties and *E*°′ values, indicating that no major changes occur in their regulating molecular factors. Most importantly, Co-WT and Co–C46AC55A can catalyze the reduction of H_3_O^+^ to H_2_, with onset potentials and overpotentials comparable to those of Co-porphyrin/polypeptide catalysts. The electrocatalytic efficiency of Co-WT and Co–C46AC55A for the development of H_2_ is slightly lower compared to that of six-coordinated aquo-His Co-Mb, although they are less affected by the presence of dioxygen.

## Abstract 2

The ability of Co-substituted derivatives of wt human neuroglobin and its C46A/C55A mutant to mediate the electrocatalytic evolution of H_2_ is shown for the first time, which compares favorably with that of Co-porphyrin/polypeptide catalysts. New and deeper insight into the electronic properties of the metal center and vibrational modes of Co-substituted heme proteins is provided, showing that heme *b* swapping with Co-PPIX does significantly modify neither the molecular factors controlling their *E*°′Co(III)/Co(II) nor their pH-dependent behavior.

## Introduction

Green hydrogen produced by water electrolysis, using electricity obtained by renewable sources, is one of the most promising alternatives to fossil fuels.^1−4^ The high overvoltage required for the discharge of H_2_ is one of the main obstacles to the large-scale production of green hydrogen since it significantly increases the energy required for the reaction compared to the theoretical value. To solve this problem, big efforts are currently underway to synthesize and produce new electrode materials, featuring a lower overvoltage for H_2_ discharge.^1,4−8^

One approach to overcome this issue could be the production of molecular hydrogen by reactions catalyzed by hydrogenases.^9,10^ Although their catalytic activity is very high,^9,11,12^ the application of these metalloenzymes in large-scale production of green H_2_ is severely hampered by their high sensitivity to ROS and O_2_, which results in their immediate inactivation.^9,13^

An alternative approach exploits cobalt-containing coordination compounds endowed with hydrogenase activity, combining a lower overvoltage for hydrogen reduction with the ability to work in the presence of dioxygen.^9,10,13,14^ Insertion of the catalytically active Co-centers into a simplified protein scaffold^15,16^ resulted in the production of several biomimetic catalysts,^11,12,15,17,18^ featuring a higher solubility and a greater catalytic efficiency in water. Alternatively, Co-substituted metalloenzymes catalyzing H_2_ production can be prepared by replacing the heme group with the corresponding Co-derivative in different heme proteins.^12,13,17,19−22^ The resulting non-natural enzymes combine a high solubility in water and a good resistance to degradation reactions with the possibility to control their catalytic efficiency for H_2_ production by selected point mutations.^12,13,17,19−22^

Recently, we have demonstrated that Co-substituted myoglobin (Co-Mb) immobilized on the surface of pyrolytic graphite electrode can efficiently perform the electrocatalytic reduction of water protons to H_2_ under anaerobic conditions, thereby providing a simple and tunable system for H_2_ production.^22^ The electrocatalytic ability of electrode-immobilized Co-Mb is affected by the ligand bound to the distal axial coordination position, as replacement of the distal water with stronger N-donor ligands hampers the catalytic reduction of H^+^ to H_2_.^22^

**Figure 1:**
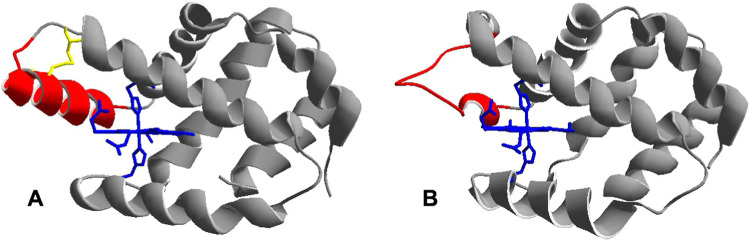
Cartoon representation of the structure of WT hNgb (4mpm.pdb, chain A) featuring the disulfide bridge between Cys46 and Cys55 (A) and of the C46G/C55S/C120S mutant of hNgb (1oj6.pdb, chain B) without the disulfide bridge between Cys46 and Cys55 (B). The heme group and iron axial ligands His64 and His96 are represented in blue, whereas Cys46 and Cys55 are depicted in yellow. The CD loop is represented in red.

Human neuroglobin (hNgb) is a monomeric globin (MW = 17 kDa), which contains a single six-coordinated heme *b*, whose axial iron coordination positions are occupied by the N_δ_ atom of the imidazole rings of His64 and His96^23−26^ (Figure 1), resulting in a sensibly reduced ability to bind exogenous ligands compared to five-coordinated globins.^23,24,27−30^

Human neuroglobin contains three cysteine residues (Cys46, Cys55, and Cys120), two of which (Cys46 and Cys55) form an intramolecular disulfide bridge under oxidizing conditions.^23−26,29,31−38^ Cleavage of the disulfide bond alters the conformation of the loop connecting the helices C and D^25,26,31,33,37^ (Figure 1) and deeply modifies the H-bonding network involving the heme propionates, without significantly altering the heme cavity and the three-dimensional structure of the remaining portions of the protein.^25,26,33^ This structural rearrangement strengthens the bond between the heme iron and the distal histidine.^25,29,33,35−38^

In this work, a combined electrochemical and spectroscopic approach has been used to perform a comprehensive characterization of the Co-substituted derivatives of WT human neuroglobin (Co-WT) and its C46A/C55A mutant (Co–C46AC55A), obtained by replacing the native heme group with cobalt protoporphyrin IX (Co-PPIX). The aim of this work is to study the ability of protein-embedded Co-PPIX to mediate the electrocatalytic reduction of water protons to H_2_ as well as to clarify the role of the metal axial ligands and of the protein environment in modulating its electronic properties and redox chemistry. hNgb was chosen because it combines a His/His axial coordination with secondary and tertiary structures closely similar to those of myoglobin, whose Co-substituted derivatives have been characterized in detail.^13,22,39−45^

We found that Co-WT and Co–C46AC55A contain a six-coordinated low-spin Co-PPIX, whose axial coordination positions are most likely occupied by the distal and proximal histidines as in the corresponding Fe species and whose electronic properties are scarcely affected by deletion of the disulfide bond. Most importantly, upon immobilization on the surface of a pyrolytic graphite electrode, both Co-WT and Co–C46AC55A catalyze the reduction of H_3_O^+^ to H_2_ with onset potentials and overpotentials comparable to those of Co-porphyrin/polypeptide catalysts. Their electrocatalytic efficiency for the development of H_2_ is slightly lower compared to the H_2_O-His-six-coordinated Co-Mb (probably due to their proposed heme His-His six coordination), but they are rather insensitive to the presence of O_2_. Both Co-WT and Co–C46AC55A feature negative *E*°′_Co(III)/Co(II)_ values, which are more than 0.2 V lower than those of Co-Mb,^13,22^ as already observed in the corresponding Fe proteins.^34^ Moreover, replacement of heme *b* with Co-PPIX does not significantly alter either the overall pattern of *E*°′_Co(III)/Co(II)_ modulation or the overall pH-dependent behavior of both Co(III) adducts compared to the ferric species,^34^ indicating that the same molecular factors are operative.

## Experimental Section

### Materials

Wild-type human neuroglobin (hNgb) and its C46A/C55A mutant were expressed in*Escherichia coli* and purified as reported previously.^35,36^ Cobalt protoporphyrin IX (Co-PPIX) was purchased from Porphyrin Systems Hombrecher e.K. (Halstenbek, Germany). Sodium monohydrogen phosphate, sodium dihydrogen phosphate, sodium chloride, potassium perchlorate, hydrochloric acid, and sodium hydroxide (Carlo Erba Reagenti) were of reagent grade. Tris (PanReac AppliChem) was of molecular biology grade. Sodium dithionite (Sigma-Aldrich) was of reagent grade. Purified water (Milli-Q Plus Ultrapure Water System coupled with an Elix-5 Kit by Millipore) was used throughout. The water resistivity was greater than 18 MΩ cm.

### Preparation of the Co-Substituted Adducts of WT and C46C55A hNgb

The Co-substituted adducts of WT hNgb and its C46AC55A mutant were obtained by inducing the release of heme *b* through acid denaturation of the iron-containing proteins, followed by reconstitution with an excess of Co-PPIX at slightly alkaline pH. Concentrated stock solutions of WT and C46C55A in Tris-HCl 50 mM plus NaCl 0.15 M pH = 8.0 were acidified to pH 2.2–2.3 by adding small aliquots of concentrated HCl and kept at rest for 2 h at 4 °C. A solution of Co(II)-PPIX was prepared by dissolving Co(III)-PPIX in NaOH 0.01 M (pH = 12.0) followed by addition of an excess of sodium dithionite. The freshly prepared Co(II)-PPIX solution was added to the Ar degassed protein solution to achieve a final stoichiometric ratio [Co(II)-PPIX]/[hNgb] of 10:1. After adjusting the pH to 8.0 by adding small amounts of concentrated NaOH solution, the obtained solution was kept at 4 °C overnight and then concentrated to a final volume of 2 mL by ultracentrifugation. To eliminate unbound Co-PPIX, the protein solution was loaded on a Sephadex G-15 gel-filtration column equilibrated with 50 mm phosphate buffer pH = 7.0 plus 0.1 M NaCl. Fractions containing Co(III)-WT and Co(III)-C46AC55A were collected and concentrated by ultrafiltration. The concentrated protein solutions were stored at 4 °C. Co-Mb was prepared as previously reported.^22^

### Electrochemical Measurements

The electrochemical measurements were carried out with a Potentiostat/Galvanostat PARSTAT (Princeton Applied Research, PAR) model 2273 in a three-electrode configuration. A 2 mm diameter edge-pyrolytic graphite disk (PGE) was used as the working electrode, while an Ag/AgCl/KCl_sat_ electrode (+0.196 V vs SHE) and a Pt wire were the reference and counter electrodes, respectively.

The PGE electrode was cleaned at the beginning of each measurement with an alumina (particle size of about 0.015 μm) water slurry on cotton wool and then sonicated in an ultrasonic pool for 1 min. Finally, the electrode was washed with nanopure water. The counter electrode was dipped for 10 min in concentrated nitric acid at high temperature and then washed with nanopure water before use.

All of the reported potentials are referred to the standard hydrogen electrode (SHE). Unless otherwise stated, voltammetric experiments were carried out under anaerobic conditions (Ar atmosphere) using 3 μM solutions of Co-Ngb (checked spectrophotometrically) in 50 mM sodium perchlorate and 5 mM phosphate buffer at different pH values.

Square wave voltammetry (SWV) experiments at different temperatures were carried out with a “nonisothermal” cell in which the reference electrode was kept at constant temperature (21 ± 0.1 °C) in a 1 M NaClO_4_/Agar salt bridge, while the half-cell containing the working electrode and the Vycor junction (from PAR) to the reference electrode was under thermostatic control.^46−48^ Formal reduction potentials *E*°′ were calculated as the semisum of the anodic and cathodic peak potentials. All of the experiments were repeated at least five times, and the *E*°′ values were found to be reproducible within ± 0.004 V. The temperature was varied from 5 to 45 °C. With this experimental configuration, the standard entropy change (Δ*S*°′_rc_) is given by
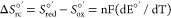
Thus, Δ*S*°′_rc_ was determined from the slope of the plot of *E*°′ versus temperature.^49−51^ The enthalpy change (Δ*H*°′_rc_) was obtained from the Gibbs–Helmholtz equation, namely, as the negative slope of the *E*°′/T versus 1/T plot.^49−51^ The nonisothermal behavior of the cell was carefully checked by determining the Δ*H*°′_rc_ and Δ*S*°′_rc_ values of the ferricyanide/ferrocyanide couple.^49,50^

The catalytic efficiency of the proteins for H_2_ development was measured under anaerobic conditions (Ar atmosphere) by evaluating the shifts of the onset potential and the H_2_ reduction current densities at different pH values and temperatures, using 50 mM sodium chloride and 5 mM tris-HCl as the base electrolyte solution. The catalytic efficiency of the proteins for H_2_ development under aerobic conditions was measured by evaluating the shifts of the onset potential and the H_2_ reduction current densities observed upon exposing the protein solution (50 mM sodium chloride and 5 mM tris-HCl as the base electrolyte) to air until full saturation with O_2_.

The absence of free CoPPIX during the electrocatalytic experiments was verified electrochemically by recording a square wave voltammetry in the potential range including the *E*°′ of the Co(III)/Co(II) redox couple of free CoPPIX before each electrocatalytic experiment.

### Electronic Absorption, MCD, and CD Measurements

All of the spectra, unless otherwise stated, have been obtained at pH 7.0 in 50 mM phosphate buffer plus 0.1 M NaCl.

The electronic absorption measurements were carried out on protein solutions freshly prepared before use at 25 °C with a Jasco J-810 spectropolarimeter using a quartz cuvette of 0.5 cm path length with a resolution of 1.0 nm, a 200 nm/min scan rate, and summing up 3 spectra to improve the signal-to-noise ratio or with a Cary60 spectrophotometer (Agilent, Santa Clara, CA) with a resolution of 1.5 nm and a 300 nm/min scan rate. In the latter case, measurements were performed in a 5 mm NMR tube or a 1 mm cuvette. In the figures, all of the spectra were normalized to the maximum intensity of the Soret band, and the intensity in the 475–700 nm region was further magnified by 7-fold for a clear visualization.

Magnetic circular dichroism (MCD) spectra were recorded using a Jasco J-810 spectropolarimeter using a quartz cuvette of 0.5 cm path length with a resolution of 1.0 nm, a 200 nm/min scan rate, and summing up 3 spectra to improve the signal-to-noise ratio. All experiments were carried out at 25 °C with protein solutions freshly prepared before use. The magnetic field for MCD measurements was provided by a Model 3470 GMW Magnet system split coil superconductivity magnet with a maximum field of 1 T (T). MCD spectra were measured in θ = mdeg and converted to Δε [M^–1^ cm^–1^ T^–1^] using the conversion factor Δε = θ/(32,980·c·d·B), where c is the protein concentration, B is the magnetic field (1 T), and d is the thickness of the sample (path length, 0.5 cm).^34,52,53^

Circular dichroism (CD) spectra were recorded using a Jasco J-810 spectropolarimeter using a quartz cuvette of 1.0 cm path length with a resolution of 1.0 nm, a 20 nm/min scan rate, and summing up 8 spectra to improve the signal-to-noise ratio. All experiments were carried out at 25 °C with protein solutions freshly prepared before use.

### Resonance Raman Measurements

Sample concentration was in the range from 15 to 25 μM. All of the spectra, unless otherwise stated, have been obtained at pH 7.0 in 50 mM phosphate buffer plus 0.1 M NaCl.

Resonance Raman (RR) spectra were obtained at room temperature (300 K) using the 404.8 nm line of a diode laser (MatchBox Series, Integrated Optics, Vilnius, Lithuania), the 441.6 nm excitation of a He–Cd laser (Kimmon IK4121R-G, Tokyo, Japan), or the 532 nm excitation of a diode laser (Cobolt Samba 300). Backscattered light was collected from a slowly rotating 5 mm NMR tube and focused into a triple spectrometer (ActonResearch, Acton, MA). The latter consists of two SpectraPro 2300i instruments that work in subtractive mode, and a SpectraPro 2500i instrument in the final stage with a grating of 3600 grooves/mm (with a nominal resolution of 1.2 cm^–1^) and 1800 grooves/mm (with a nominal resolution of 4 cm^–1^) and equipped with a liquid-nitrogen-cooled CCD detector. The spectra are obtained with the 3600 grooves/mm grating unless indicated.

For the low-temperature experiments, a 60 μL droplet of the sample was put in a 1.5 cm diameter quartz crucible inside a THMS600 cryostat (Linkam Scientific Instruments, Surrey, UK) and frozen to 80 K. To prevent sample denaturation or photoreduction, the laser position on the sample was changed frequently.

The RR spectra wavenumbers were calibrated to an accuracy of 1 cm^–1^ for intense isolated bands using indene, carbon tetrachloride, and DMSO as standards. The power on the sample was 4, 6, and 45 mW at 404.8, 441.6, and 532 nm excitation wavelengths, respectively. To improve the signal-to-noise ratio, the RR measurements were repeated multiple times under the same conditions and summed, only if there were no spectral differences. The RR spectra were baseline-corrected and those obtained with the Soret excitation wavelengths, normalized in the high-wavenumber region to the intensity of the ν_4_ band, and in the low-wavenumber region to the intensity of the ν_8_ band. All of the RR spectra obtained at 532 nm were normalized to the intensity of the ν_19_ band.

The curve-fitting analysis of the spectra was performed by using a spectral deconvolution program (LabCalc; Galactic Industries, Salem, NH) with a Lorentzian line shape to determine the peak wavenumbers, bandwidth (full width at half-maximum), and intensities, with an accuracy of 1 cm^–1^ for wavenumbers and widths.

### Effect of pH on the Spectroscopic and Redox Properties of Co-WT and Co–C46C55A

The pH-induced changes in the absorbance of the Soret band for Co(III)-WT and Co(III)-C46AC55A were interpolated using eqs 1 and 2, respectively,^34^ whereas the changes of the intensity of the second derivative of the Soret band, of the peak-to-trough distance of the MCD Soret signal, and of the molar ellipticity at 222 nm at different pH values for both Co(III)-substituted proteins could be fit using eqs 3, 4, and 5, respectively.^34^
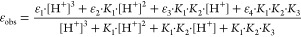
1
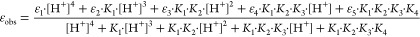
2
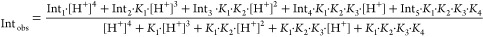
3
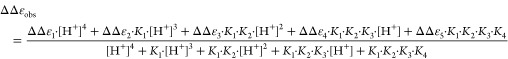
4
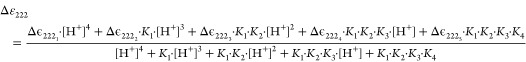
5

In both cases ε_obs_, Int_obs_, ΔΔε_obs_, and Δε_222_ correspond to the molar extinction coefficient at 423 nm (Co(III)-WT) and 422 nm (Co(III)-C46AC55A), the intensity of minimum of the second derivative of the Soret band, the peak-to-trough distance of the MCD Soret signal, and the difference between the extinction coefficient for the left- and right-hand circular polarized light at 222 nm measured at different pH values, respectively, while ε_n_, Int_n_, ΔΔε_n_, and Δε_222__n_ correspond to the molar extinction coefficient at 423 nm (Co(III)-WT) and 422 nm (Co(III)-C46AC55A), the intensity of minimum of the second derivative of the Soret band, the peak-to-trough distance of the MCD Soret signal, and the difference between the extinction coefficient for the left- and right-hand circular polarized light at 222 nm of each protein conformer observed at different pH values, starting from that stable at lower pH values, respectively.

The pH dependence of *E*°′ of Co-WT and Co–C46AC55A immobilized on PGE could be fitted using the following three-equilibrium Clark Dutton equation:^34,54,55^
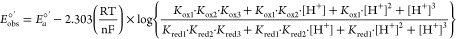
6in which *E*°′_obs_ is the measured potential at any given pH; *E*°′_a_ is the reduction potential of the fully protonated form of Co-WT and Co–C46AC55A; and *K*_ox1_, *K*_ox2_, *K*_ox3_ and *K*_red1_, *K*_red2_, and *K*_red3_ are the acid–base equilibrium constants for the Co(III) and Co(II) forms of Co-WT and Co–C46AC55A, respectively.

## Results and Discussion

### Redox Properties of Co-WT and Co–C46AC55A at Neutral pH

**Figure 2:**
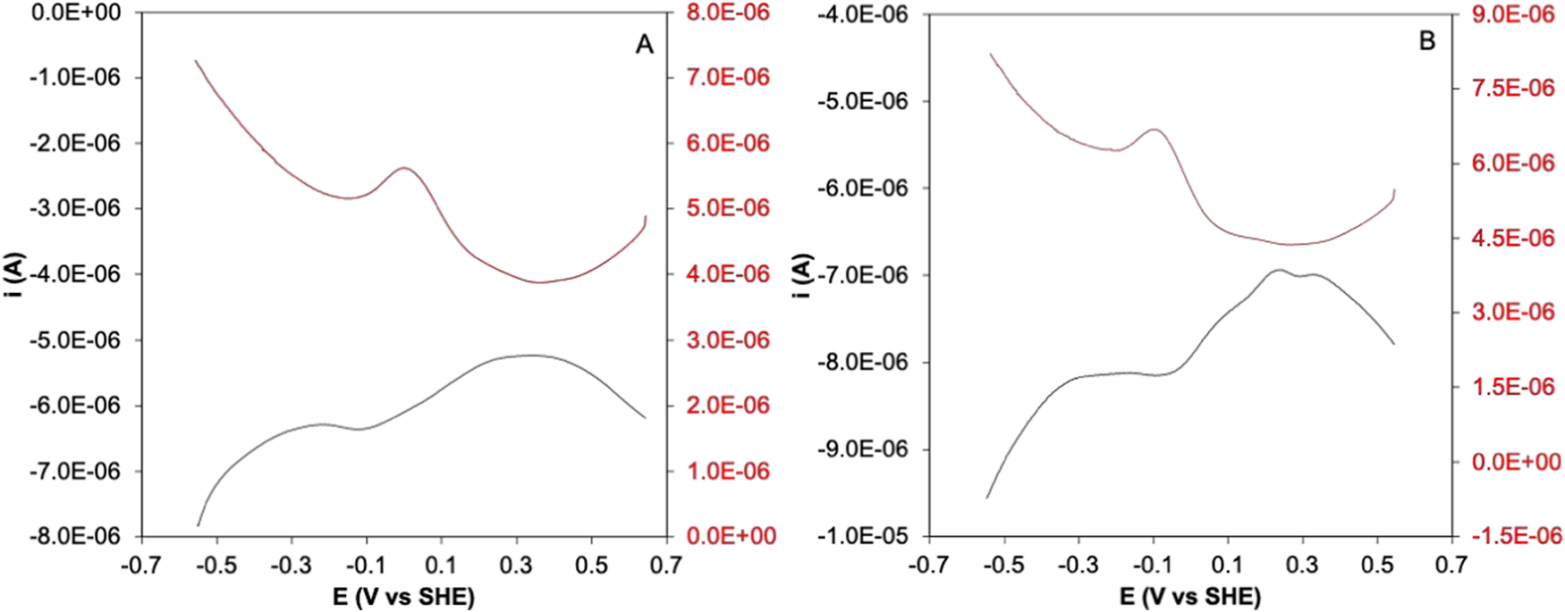
Square wave voltammetries of 3 μM Co-WT (A) and Co–C46AC55A (B) on PGE in 5 mM phosphate buffer plus 50 mM KClO_4_ at pH 7.0, *T* = 20 °C, frequency of 5 Hz, pulse amplitude 0.025 V, Ar atmosphere. Cathodic and anodic scans are reported as red and black lines, respectively.

Figure 2 shows the square wave voltammetries obtained for Co-WT (A) and Co–C46AC55A (B) at pH 7.0 and *T* = 20 °C on PGE under anaerobic conditions. The corresponding cyclic voltammetries show weak and unresolved signals and therefore were not considered.

One single cathodic peak and its anodic counterpart are observed; both signals are stable upon several SWV scans and indicate a quasi-reversible electrochemical process. Upon previous immersion of the electrode into the protein solution, the signals are also observed when the SWVs were recorded in the base electrolyte solution but disappeared after the third voltammetric cycle. As a consequence, all measurements were performed with the electrode immersed in the Co-Ngb solution. The observed signals can therefore be confidently associated with the reversible reduction/oxidation of the Co(III)/Co(II) couple of Co-WT and Co–C46AC55A immobilized on the electrode surface. Accordingly, the *E*°′ values were calculated as the semisum of the cathodic and anodic peaks (Table 1). The barely detectable shoulder at about 0 V can be ascribed to a small amount of free Co-PPIX. The reliable characterization of the redox reactivity of both proteins under freely diffusing conditions was prevented by the extremely low quality of their electrochemical response.

**Table 1:** Reduction Potentials (*E*°′), Standard Reduction Enthalpy (Δ*H*°′_rc_) and Entropy (Δ*S*°′_rc_), and the Corresponding Contributions to *E*°′_Co(III)/Co(II)_ for Co-WT and Co–C46AC55A Immobilized on PGE at pH 7.0, Base Electrolyte 5 mM Phosphate Buffer Plus 50 mM KClO_4_

protein	*E*°′^a^^,^^b^ (V)	Δ*S*°′_rc_^a^ (J·mol^–1^·K^–1^)	Δ*H*°′_rc_^a^ (KJ·mol^–1^)	*T*·Δ*S*°′_rc_/F^a^^,^^b^ (V)	–Δ*H*°′_rc_/F (V)
Co-WT	–0.049	+114	+38	0.346	–0.396
Co–C46AC55A	–0.062	+129	+44	0.392	–0.453

a Average errors on *E*°′, Δ*H*°′_rc_, and Δ*S*°′_rc_ are ±0.004 V, ±2 kJ·mol^–1^, ±8 J·mol^–1^·K^–1^, respectively.

b At 20 °C.

Both Co-WT and Co–C46AC55A feature negative *E*°′ values, which are more than 0.2 V lower (−0.219 ≤ Δ*E*°′ ≤ −0.267 V) than those of Co-Mb and its adducts with imidazole and NH_3_ in similar experimental conditions.^22^ This is consistent with the behavior of the corresponding Fe proteins, whose *E*°′ is about 0.2 V lower than that of Mb.^34^ Moreover, deletion of the disulfide bridge induces a slight decrease of the reduction potential of the Co(III)/Co(II) and Fe(III)/Fe(II) couples.^34^ Since the spectroscopic data indicate that the coordinative properties of the metal center in Co-WT and Co–C46AC55A are largely conserved (see below), the origin of this effect should be sought outside the first coordination sphere.

The enthalpy (Δ*H*°′_rc_) and entropy (Δ*S*°′_rc_) changes accompanying the reduction of Fe^3+^ to Fe^2+^ provide information on the molecular factors modulating the *E*°′ of heme proteins.^34,47,51,56−62^ Δ*H*°′_rc_ is mainly influenced by the structural and electronic properties of the metal center (number and type of ligands, coordination geometry) and by its electrostatic interactions with the charges and dipoles of the polypeptide chain and the solvent, while Δ*S*°′_rc_ is mainly related to reduction-induced solvent reorganization effects.^34,51^

The temperature profiles of the *E*°′_Co(III)/Co(II)_ for PGE-immobilized Co-WT and Co–C46AC55A are shown in Figure S1, and the corresponding Δ*S*°′_rc_ and Δ*H*°′_rc_ values are reported in Table 1. Since both proteins feature positive Δ*H*°′_rc_ and Δ*S*°′_rc_ values, their negative *E*°′ values result from a larger enthalpic contribution (−Δ*H*°′_rc_/F), which prevails over a smaller entropic contribution (*T*·Δ*S*°′_rc_/F), favoring Co(III) reduction (Table 1). The same behavior has been previously observed for Fe-hNgb and many other heme proteins.^34,47,51,57−62^ Removal of the disulfide bridge exerts a rather limited effect on the reduction thermodynamics of the metal center (Table 1) as observed for Fe-containing neuroglobins.^34^

Hence, replacement of heme *b* with Co-PPIX results in different reduction thermodynamics, but it does not significantly alter the H–S interplay that controls the *E*°′ value in hNgb.^34^ This suggests that the redox reactivity of the Fe(III)/Fe(II) and Co(III)/Co(II) couples in WT and C46C55A is controlled by the same molecular factors.^34^ Indeed, the positive Δ*S*°′_rc_ values of Co-WT and Co–C46AC55A fit with a reduced solvent ordering upon Co(III) reduction, owing to the decreased electrostatic interaction of the water molecules within the hydration sphere with the Co(II) protein as compared with the Co(III) species (Δ*S*°′_rc(solv)_ > 0).^51,56−62^ The increased Δ*S*°′_rc_ of Co–C46AC55A compared to Co-WT indicates that cleavage of the Cys46/Cys55 disulfide bridge slightly enhances the reduction-induced solvent reorganization within the hydration sphere of the protein.^35,54^

As the entropy and enthalpy changes associated with solvent reorganization effects exactly offset,^34,51^ the measured Δ*G*°′_rc_ of the reduction reaction (= −nFE°′) depends exclusively on the protein-based contribution to reduction enthalpy.^34,51^ Therefore, the positive enthalpic intrinsic contribution to *E*°′ (Δ*H*°′_rc(int)_ = Δ*G*°′_rc_ = −nFE°′) of Co-WT and Co–C46AC55A is consistent with the selective stabilization of the Co(III) species by strong axial donors, such as two axial histidines (see below).^34,51^ On the other hand, the limited differences existing between the *E*°′ of Co-WT and Co–C46AC55A indicate that cleavage of the Cys46/Cys55 disulfide bond does not significantly alter the ligand binding interactions nor the electrostatics at the metal sites, as in the case of the corresponding Fe proteins.^34^

### Electrocatalytic H_2_ Evolution Mediated by Co-WT and Co–C46AC55A

**Figure 3:**
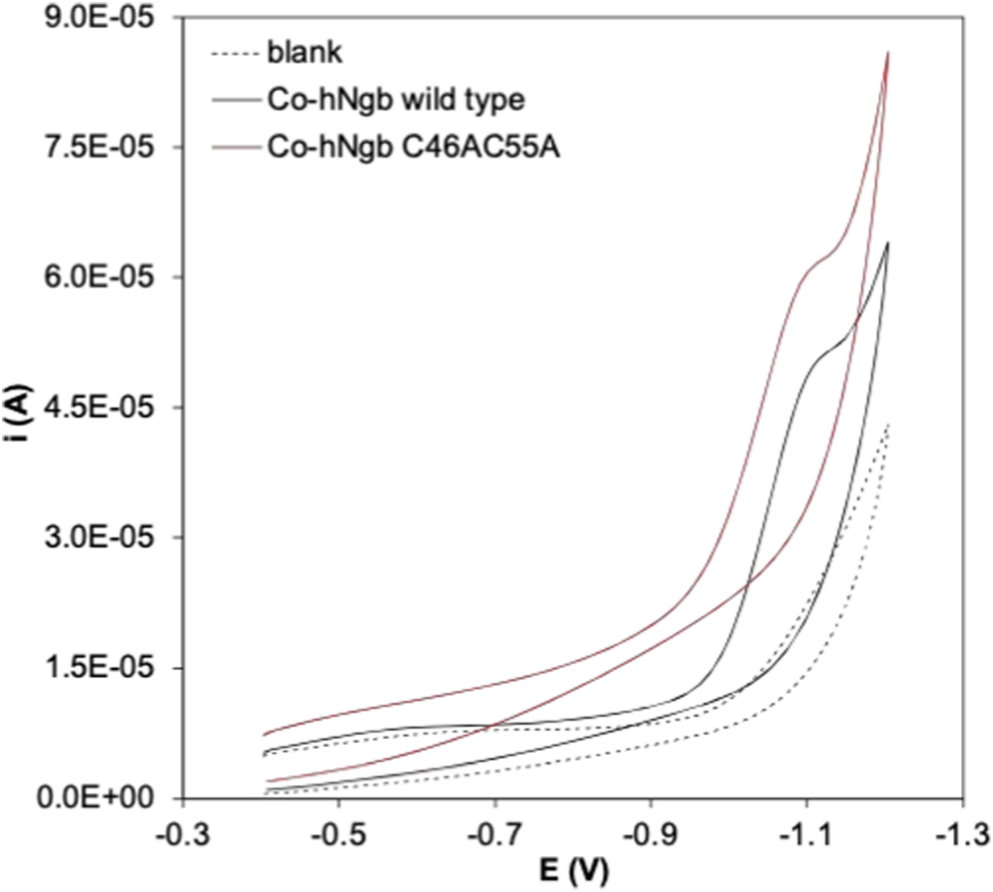
Cyclic voltammetry of 3 μM Co-WT (black), Co–C46AC55A (red), and on bare PGE (dotted), *T* = 20 °C, pH 7.0, Ar atmosphere. Base electrolyte 5 mM tris-HCl buffer plus 50 mM NaCl.

The ability of Co-WT and Co–C46AC55A to catalyze the electrochemical reduction of H_3_O^+^ to H_2_ under anaerobic conditions was studied by cyclic voltammetry (*CV*) in the negative potential window (between −0.4 and −1.2 V vs SHE), where H_2_ formation occurs (Figure 3).

At neutral pH and room temperature (*T* = 20 °C), the onset potential of the signal associated with the reduction of H_3_O^+^ to H_2_ on bare PGE is observed at about −1.04 V (vs SHE). In the presence of 3 μM Co-WT or Co–C46AC55A under the same experimental conditions, a well-shaped sigmoidal cathodic signal can be observed at less negative potentials, which can be assigned to the electrocatalytic hydrogen evolution mediated by the protein adsorbed on the surface of the PGE (Figure 3). The *C* overpotentials, calculated according to ref (18), resulted to be 0.623 ± 0.005 and 0.641 ± 0.005 V (*T* = 25 °C, pH = 7.0) for Co-WT and Co–C46AC55A, respectively (Table 2). Possible contributions from free Co-PPIX can be ruled out since its absence during the electrocatalytic experiments was verified electrochemically (see the Experimental Section) and the voltammetric signal arising from the electrocatalytic hydrogen evolution by Co-PPIX occurs at different potentials compared to Co-WT and Co–C46AC55A (see Figure S2).

**Table 2:** Catalytic Parameters of H_2_ Evolution by Co-WT and Co–C46AC55A and Selected Co-Porphyrin/Peptide Electrocatalysts

	pH	onset potential^a^ (V)	overpotential^a^ (V)
Co-WT^b^^,^^c^	7.0	–0.97	0.623
Co–C46AC55A^b^^,^^c^	7.0	–0.95	0.641
Co-MP11^d^	7.0	–0.98	0.852
MC6*^e^	6.5	–0.87	0.680
Ht-CoM61A^f^	7.0	–1.07	0.830
Co-Mb^g^	7.2	–0.92	0.608
Co-Mb+Imidazole^g^	7.2	–0.94	0.622
Co-Mb+NH_3_^g^	7.2	–0.98	0.646

a Calculated as reported in ref (18).

b at *T* = 25 °C and pH = 7.0.

c associated error ± 0.005 V.

d from ref (12).

e from ref (18).

f from ref (19).

g from ref (22).

The observed onset potentials (Table 2) are about 0.07 and 0.09 V more positive, respectively, than that observed in the absence of the protein and approximately 0.03–0.01 V more positive than that reported for water-soluble Co-porphyrins.^22,63^ On the other hand, they are 0.05–0.07 V more negative than that observed for Co-Mb immobilized on PGE electrode in similar experimental condition and comparable to those of its imidazole and NH_3_ derivatives.^22^ Accordingly, the overpotentials of Co-WT and Co–C46AC55A (0.623 and 0.641 V, respectively) are 0.03–0.04 V higher than those measured for Co-Mb and similar to those of its adducts with nitrogenous axial ligands.^22^ In addition, the onset potentials and overpotentials of Co-WT and Co–C46AC55A are similar to or even lower than those of Co-porphyrin/polypeptide used as electrocatalysts.^12,18,19^ Therefore, it appears that both Co-WT and Co–C46AC55A can catalyze the reduction of H_3_O^+^ to H_2_. Overall, their electrocatalytic behavior is similar to that of Co-Mb and its imidazole and NH_3_ adducts,^22^ suggesting that the catalytic production of H_2_ results from a mechanism which combines the reduction of Co(I) to Co(0) with a concerted PCET (proton-coupled electron transfer), as previously proposed for Co-Mb.^12,13,18,19^ Nevertheless, the likely His-His six coordination of Co-hNgb induces a rather limited decrease of the electrocatalytic ability for the development of H_2_ compared to H_2_O-His-axially coordinated Co-Mb.

The values of the onset potential of both Co-WT and Co–C46AC55A show a marked pH dependence (Figures S3),^17,64−66^ indicating that in acidic condition, the former is more efficient in lowering the onset potential for H_2_ production.

The catalytic current density *j*_cat_ for PGE-immobilized Co-WT and Co–C46AC55A increases from pH 11 to 7 with a sigmoidal behavior (Figure S4), characterized by a slightly higher limiting current density for the WT protein (about 16%). From pH 3 to 7, both proteins show higher catalytic current densities and therefore better catalytic efficiencies, in agreement with the pH dependence of the onset potential. Below pH 3, the electrocatalytic signal gradually changes and disappears, indicating progressive protein denaturation. The pH dependence of the catalytic current density *j*_cat_ for Co-WT and Co–C46AC55A is different from that of Co-Mb.^22^ Similar behaviors have been already reported for other proteins or bioinspired systems catalyzing hydrogen evolution electrochemical reactions.^17,65,66^ The slightly higher catalytic efficiency of Co-WT in acid conditions is consistent with the lower structural stability of its metal center below pH 4, indicated by the different spectroscopic behavior of the two Co-adducts (see below), which could result in an enhanced solvent accessibility of the metal center and/or in a different protein conformation, yielding a more catalytically active prosthetic group.

**Figure 4:**
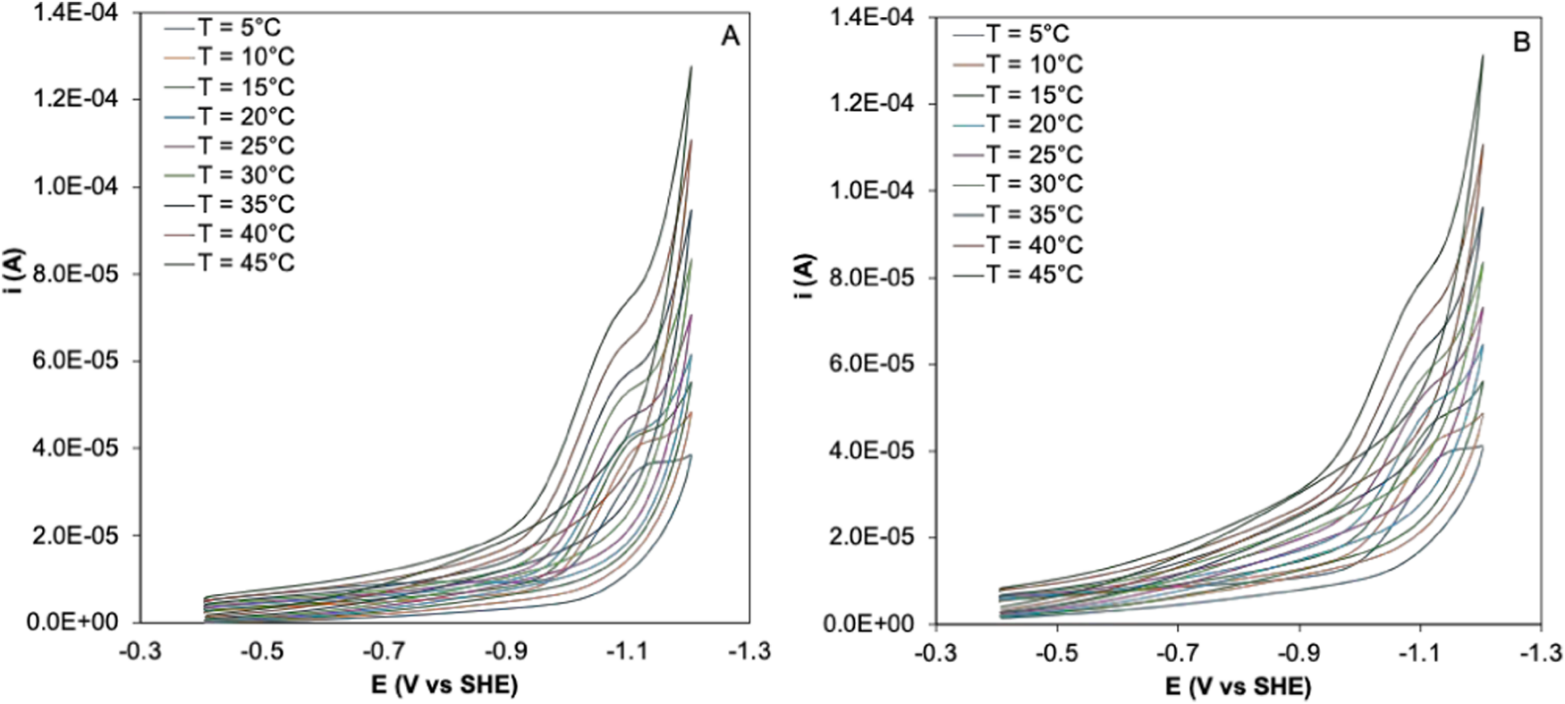
Cyclic voltammetries recorded at different temperatures for 3 μM Co-WT (A) and Co–C46AC55A (B) at pH 7.0. Base electrolyte 5 mM tris-HCl buffer plus 50 mM NaCl, Ar atmosphere.

With increasing temperature, the signal due to the electrocatalytic reduction of H_3_O^+^ to hydrogen progressively shifts to less negative potentials (from about −1.16 and −1.18 V at 5 °C to −1.10 and −1.11 V at 45 °C for Co-WT and Co–C46AC55A, respectively) and the catalytic current density *j*_cat_ increases (Figures 4 and S5).

**Figure 5:**
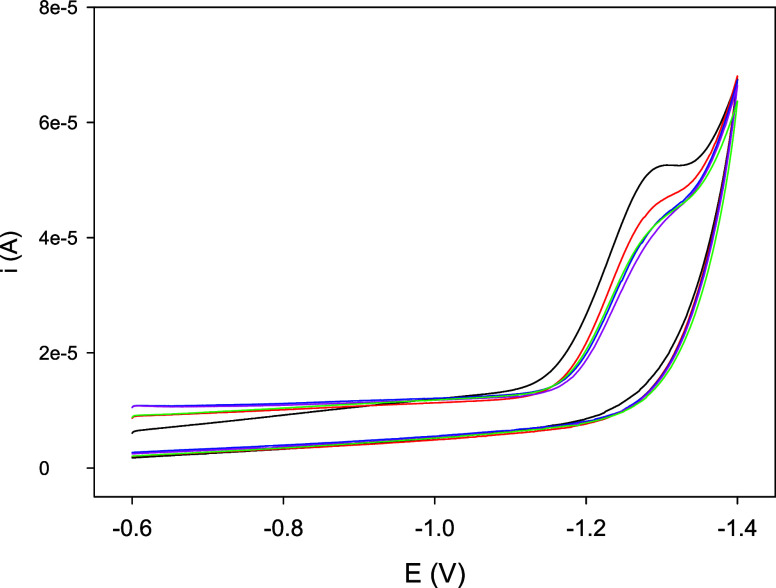
Cyclic voltammetries of 3 μM Co-WT at pH 7.0 under anaerobic conditions (black) and after 5 (red), 10 (blue), 15 (pink), and 40 (green) minutes of exposition to atmospheric O_2_. Base electrolyte 5 mM tris-HCl buffer plus 50 mM NaCl.

The effect of O_2_ on the ability of Co-WT and Co–C46AC55A to catalyze the electrochemical reduction of H_3_O^+^ to H_2_ was tested by performing electrocatalytic experiments starting from anaerobic conditions and then gradually exposing the protein solution to air and measuring the catalytic curve over time until the solution is saturated with O_2_. The *CV* curves obtained for Co-WT are reported in Figure 5 and show that under O_2_ saturation conditions the protein is still catalytically active. The onset potential is barely affected by O_2_ (−0.98 vs −0.97 V under anaerobic conditions), whereas the catalytic current is lower compared to anaerobic conditions. Similar results were obtained for Co–C46AC55A. Hence, it appears that the likely His-His six coordination prevents the Co(II) formed by electrochemical reduction from being coordinated and reoxidized to Co(III) by O_2_, making it available for reductive electrocatalysis of H_3_O^+^ to H_2_ and rendering the electrocatalytic efficiency of Co-WT and Co–C46AC55A rather insensitive to the presence of O_2_.

### Spectroscopic Properties of Co(III)-WT and Co(III)-C46AC55A

**Figure 6:**
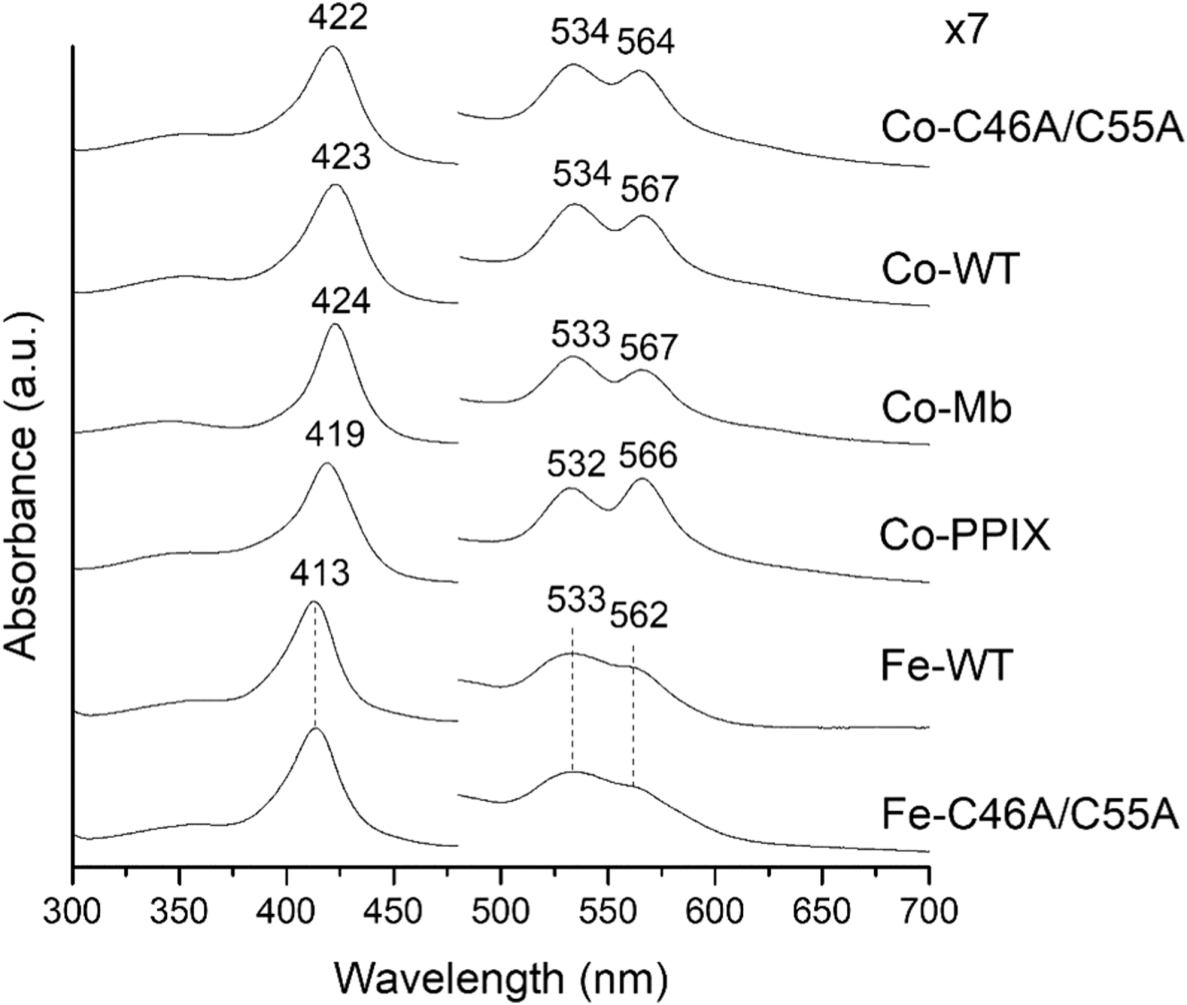
UV–vis spectra of Co(III)-C46AC55A (Co–C46A/C55A), Co(III)-WT (Co-WT), Co(III)-Mb (Co-Mb), Co(III)-PPIX (Co-PPIX), Fe(III)-WT (Fe-WT), and Fe(III)-C46C55A (Fe–C46A/C55A).

The UV–vis electronic absorption spectra of Co(III)-WT and Co(III)-C46AC55A are reported in Figure 6, together with those of Fe(III)-WT, Fe(III)-C46AC55A, Co(III)-Mb and Co(III)-PPIX. As other neuroglobins,^67,68^ the UV–vis electronic absorption spectra of Fe(III)-WT and Fe(III)-C46C55A are characteristic of a six-coordinated low-spin (6cLS) ferric iron porphyrin (Soret band at 413 nm, Q bands at 533 and 562 nm). The Co(III)-PPIX in solution shows a red shift of the overall spectrum, which becomes even larger upon binding to the Ngb scaffold. Indeed, the electronic spectrum of Co(III)-WT features a Soret band at 423 nm and Q bands at 534 and 567 nm (Table 3) and the C46AC55A double mutation induces a 1–3 nm blue shift of both the Soret and the Q bands (Figure 6 and Table 3). At variance with free Co(III)-PPIX, the β band is more intense than the α band upon binding to the protein scaffold. Both Co(III)-WT and Co(III)-C46AC55A contain a low-spin six-coordinated Co(III), in agreement with other Co-substituted heme proteins studied so far, which invariably contain a low-spin (*S* = 0) six-coordinated Co(III), even in the presence of a distal coordinating H_2_O ligand (e.g., Co(III)-Mb in Figure 6).^20,22,39−42,45,69−75^

**Table 3:** Wavelengths (λ, nm) of the Relevant Spectral Bands in the UV–Vis Spectra, the Second Derivative UV–Vis Spectra, and MCD Spectra of Co(III)-WT, Co(III)-C46AC55A, and Co(III)-PPIX at pH 7.0

	UV–vis			2nd der	MCD							
	Soret	β	α	Soret	Soret max	Soret min	Soret zerocross	β max	β min	α max	α min	α zerocross
Co(III)-WT	423	534	567	425	414	435	425	522	541	559	578	568
Co(III)-C46AC55A	422	534	564	423	411	432	422	521	538	556	576	565
Co(III)-PPIX	419	532	566	417	412	427	419	517	536	557	571	564
Co(III)-Mb^a^	424	533	567	424	418	432	426	525	540	561	578	569
Co(III)-Mb + imidazole^a^	425	535	569	426	421	434	430	524	542	560	576	569
Co(III)-Mb + NH_3_^a^	425	534	570	425	421	434	429	524	538	560	579	568

a from ref (22).

**Figure 7:**
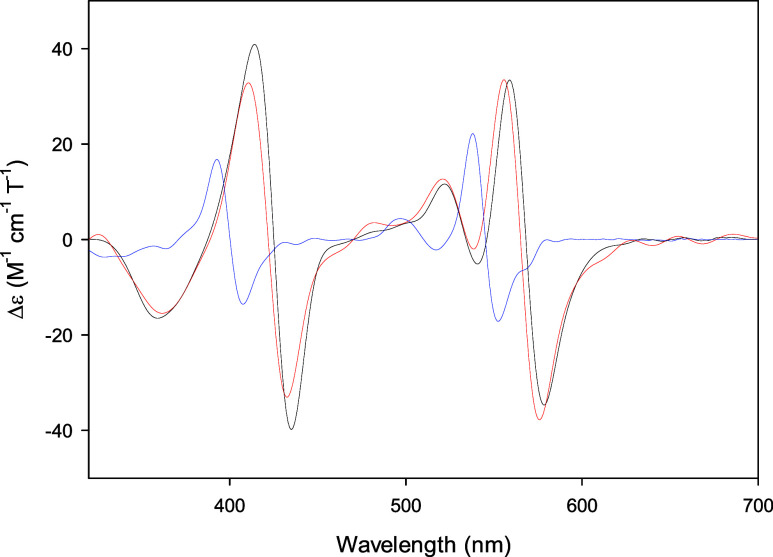
MCD spectra of Co(III)-WT (black), Co(III)-C46AC55A (red), and Co(III)-PPIX (blue) at pH 7.0. Protein and Co(III)-PPIX concentrations are 10 and 20 μM, respectively.

The 6cLS species is confirmed by the corresponding MCD spectra (Figure 7), which show two intense derivative-shaped signals in correspondence with the Soret and α/β bands (Table 3). Incorporation of Co(III)-PPIX into WT scaffold induces a sizable red shift of both signals as well, and the C46AC55A double mutation induces a limited blue shift compared to Co(III)-WT (Figure 7 and Table 3). The MCD spectra of Co(III)-WT and Co(III)-C46AC55A are similar to those of low-spin Co(III)-Mb with different axial ligands,^22^ confirming that the MCD spectra of Co(III)-globins are similar to those of 6cLS ferrous heme proteins,^76,77^ in agreement with the common 3d^6^ electronic configuration of low-spin Co(III) and Fe(II).^22,39,40^

The spectroscopic properties of Co-substituted heme proteins are much less sensible to the nature and the strength of the axial ligands, compared to the corresponding Fe proteins.^20,22,39−42,45,69−75^ However, the similarities between the UV–vis and MCD spectra of Co(III)-WT and Co(III)-C46AC55A above 500 nm with those of the NH_3_ and imidazole adducts of Co(III)-Mb^22^ can be ascribed to the presence of two nitrogenous axial ligands. This is consistent with coordination of the proximal and distal His to Co(III)-PPIX, suggested by the redox thermodynamics of the Co(III)/Co(II) redox couple.

The far-UV CD spectra of Co(III)-WT and Co(III)-C46AC55A (Figure S6) show two minima at 210 and 222 nm, whose depth is reversed compared with the ferric proteins, suggesting that replacement of the native heme with Co-PPIX somewhat affects the secondary structure of neuroglobin.

**Figure 8:**
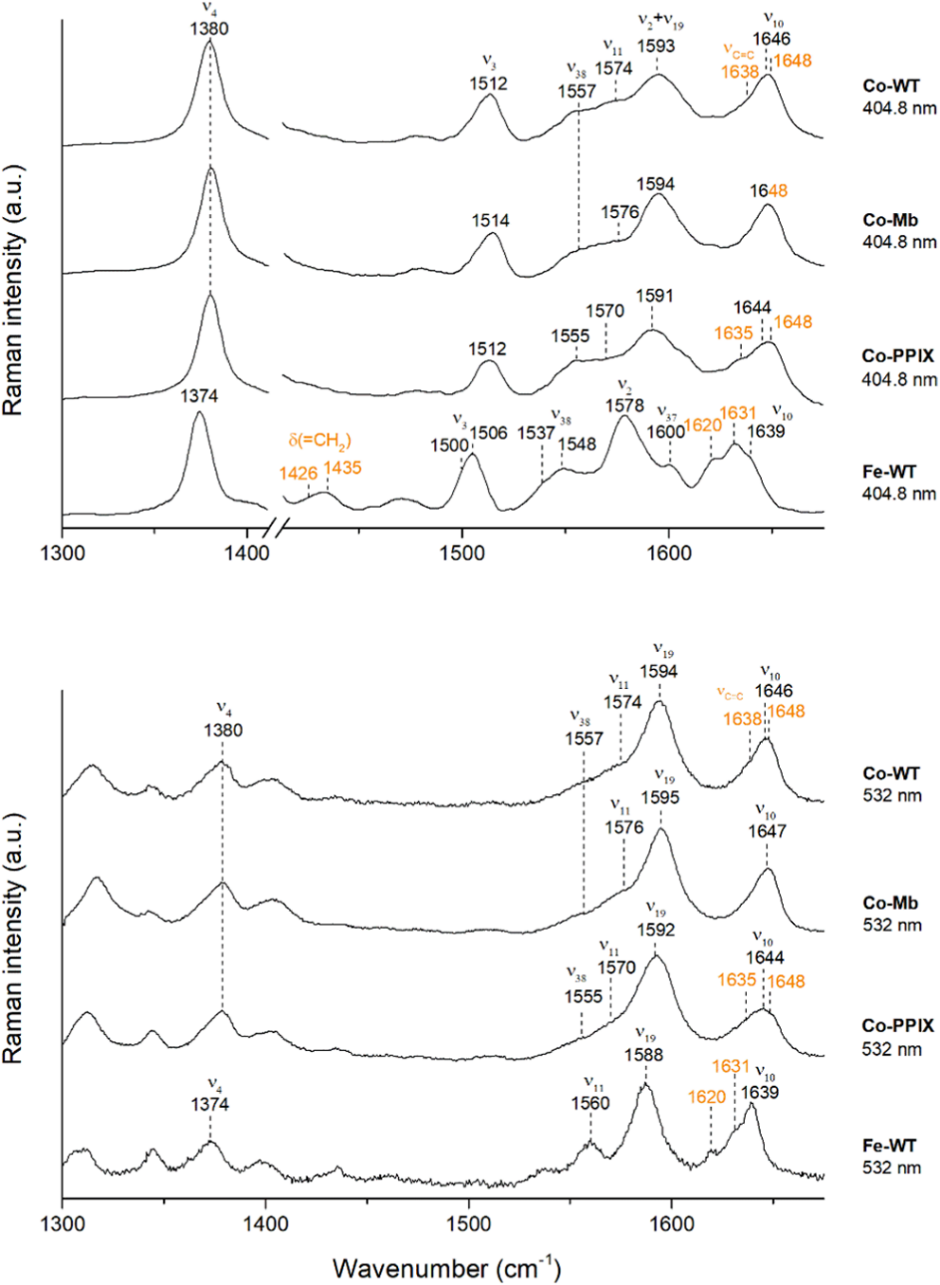
RR spectra of Fe(III)- and Co(III)-WT (Fe-WT and Co-WT), together with Co(III)-PPIX (Co-PPIX) and Co(III)-Mb (Co-Mb) at pH 7.0, taken with different laser excitation wavelengths with a 1800 grooves/mm grating. The vinyl stretching modes are reported in orange.

**Figure 9:**
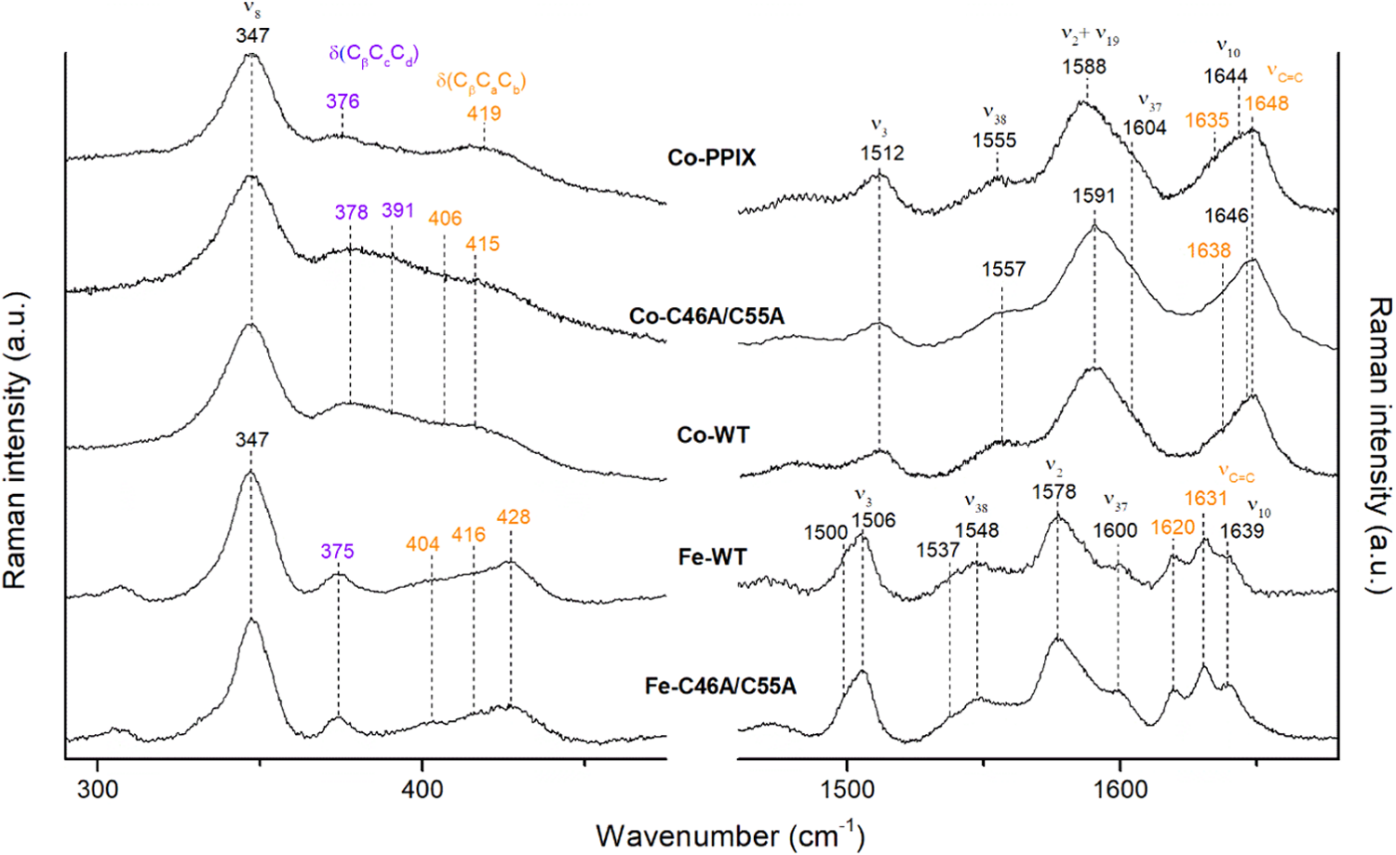
Comparison of the RR spectra of Co(III)-PPIX (Co-PPIX), Co(III)-C46AC55A (Co–C46A/C55A), and Co(III)-WT (Co-WT) upon 441.6 nm excitation with the corresponding ferric proteins (Fe-WT and Fe–C46A/C55A) obtained with 404.8 nm at pH 7.0. The spectra of Fe–C46A/C55A are obtained with a 1800 grooves/mm grating. Propionate bending, and vinyl bending and stretching modes are reported in purple and orange, respectively.

RR spectroscopy is a powerful technique to study metal porphyrins and their interactions with proteins. The high-wavenumber region (between 1300 and 1700 cm^–1^) gives information on the coordination and spin states of the metal, which are correlated to the wavenumbers of the so-called core size marker bands.^78^ The spectra of wild-type Fe(III)-hNgb in Figures 8 and 9 are identical to those of murine Ngb, characterized by a double set of core size marker bands (e.g., ν_3_), due to the presence of two 6-coordinated low-spin species, associated with the heme rotational disorder.^68,79^ In fact, in Fe-Ngbs, the heme exists in two possible insertion orientations (canonical and reversed conformers), resulting from the rotation of the heme group by 180° about the α,γ-meso axis in the protein pocket.^68,79^

Although there is extensive literature on protein in complex with iron porphyrins, systematic studies on their cobalt counterparts are still lacking. In fact, after the early work including oxy and deoxy Mb containing cobalt-porphyrins as the prosthetic groups^80^ and a more general characterization of metal reconstituted porphyrin model compound,^81^ recently, only the effect of Co on ligand binding of globins, mainly O_2_, has been characterized.^82−85^

The RR spectra of both Co(III)-Ngb and its mutant Co(III)-C46AC55A are identical. By exciting both proteins at 404.8 nm and 441.6 nm (on the blue and the red side of the Soret band, respectively) and at 532 nm (on the Q bands), it is possible to highlight different types of vibrational modes (Figures 8 and 9). The full assignment of the spectra, confirmed by the RR spectra in polarized light (Figure S7), has been obtained. The excitation wavelength at 441.6 nm, gives rise to a spectrum of the Co(III)-WT typical of those obtained in resonance with the Soret band, with enhancement of the A_1g_ totally symmetric modes (ν_4_, ν_3_, ν_2_).^86^ On the other hand, the RR spectra obtained with excitation at 404.8 nm, on the blue side of the Soret band, clearly show the enhancement of the B_1g_ (ν_11_ and ν_10_ at 1574 and 1646 cm^–1^, respectively) and A_2g_ (ν_19_ at 1594 cm^–1^) non-totally symmetric vibrational modes (Figures 8, 9, and S7), differently from Fe-Ngb.^87^ The same result is observed in Co-Mb and has been reported for horseradish peroxidase type c (HRPC) iron derivatives as well.^88^ The spectra obtained upon excitation in resonance with the Q bands (532 nm) (Figure 8) are dominated mainly by the B_1g_ and A_2g_ non-totally symmetric modes, which are effective in vibronic mixing of the Soret and Q bands as for their iron corresponding proteins.^89−91^

The RR spectra of the Co(III)-WT and its mutant are very similar to those of Co(III)-PPIX and resemble closely those of Co(III)-Mb, whose distal axial coordination position is occupied by a water molecule.^22^ This result is in agreement, however, with the X-ray structure of Co(III)-Mb,^45^ which shows that the cobalt-N(His93) proximal bond distance is shorter than in the Fe(III) protein (2.06 vs 2.17 Å), with a slightly larger hydrogen bond distance between Nε of distal His64 and the coordinated water^45^ (2.87 vs 2.65 Å). Therefore, the RR spectra clearly show that the core size marker bands are not affected by the nature of the Co(III)-distal ligand, different from the corresponding iron proteins.

In addition to the core size marker bands, the ν_C=C_ vinyl stretching modes, observed at 1620 and 1631 cm^–1^ in ferric Ngb, shift to higher wavenumbers in the Co(III) derivatives, indicating a conformational rearrangement. One ν_C=C_ vinyl stretching modes is observed at 1648 cm^–1^ in all investigated compounds and the other is at 1635 cm^–1^ for Co(III)-PPIX and at 1638 cm^–1^ for Co(III)-WT and its mutant, while both modes overlap at 1648 cm^–1^ for Co-Mb (Figures 8, 9, and S7). Figure S8 shows the spectral deconvolution of Co(III)-WT in order to highlight the presence of a weak band at 1638 cm^–1^ assigned to the second vinyl stretching mode.

A rearrangement of the peripheral substituents of the porphyrin upon Co insertion is confirmed by the RR spectra in the low-wavenumber region, where the bending modes of the propionate (in the 360–390 cm^–1^ region) and vinyl groups (in the 400–440 cm^–1^ region) of the porphyrin are observed (Figure 9). The wavenumbers of the propionate bending modes δ(C_β_C_c_C_d_) are sensitive to the H-bonding interactions with the polar residues of the active site.^92,93^ The higher their wavenumber, the stronger the H-bond with the protein moiety. The vinyl bending modes δ(C_β_C_a_C_b_) are correlated to the different orientation of these peripheral groups inside the protein cavity.^94^ Unfortunately, the connection between the torsional angle and the wavenumbers of the bending modes is less conclusive than for the corresponding stretching vibrations, due to the complex composition of the modes, involving the pyrrole deformation vibrations.^95^

Unlike Fe(III)-WT, characterized by propionate bending modes overlapping at 375 cm^–1^ as in murine Ngb,^68^ in the low-wavenumber region of the Co(III)-WT and Co(III)-C46AC55A spectra, the propionate bending modes are observed at about 378 and 391 cm^–1^. Since in Co(III)-PPIX only a broad band at 376 cm^–1^ is clearly detected, the changes are consistent with the formation of H-bond interactions between the protein matrix and propionates 6 and 7 with different strengths upon complexation of the Co(III)-PPIX with the protein. Similarly, the vinyl bending modes result in a single broad band at 419 cm^–1^ for Co(III)-PPIX, while Co(III)-WT and Co(III)-C46AC55A display modes at 406 and 415 cm^–1^, even at 80 K (data not shown). Figure S8 shows the deconvoluted spectra in the low-wavenumber region of Co(III)-WT. The absence of the double set of the core size marker bands and the presence of only two vinyl bending modes (with respect to the three bending modes at 404, 416, and 428 cm^–1^ observed for the WT in Figure 9 and as detailed in ref (79)) clearly indicate that in the Co(III) proteins the heme rotational disorder disappears and probably only the canonical orientation, which represents the most abundant species in native heme proteins, is available. Interestingly, deletion of the C46A/C55A disulfide bridge does not significantly affect either the electronic absorption spectrum or the molecular vibrations in the RR spectra. The ferric WT protein and the C46A/C55A mutant show almost identical spectra, and a similar result is observed for the corresponding Co(III) derivatives in both the high- and low-wavenumber regions (Figure 9).

### Spectroscopic Properties of Co(II)-WT and Co(II)-C46AC55A

**Figure 10:**
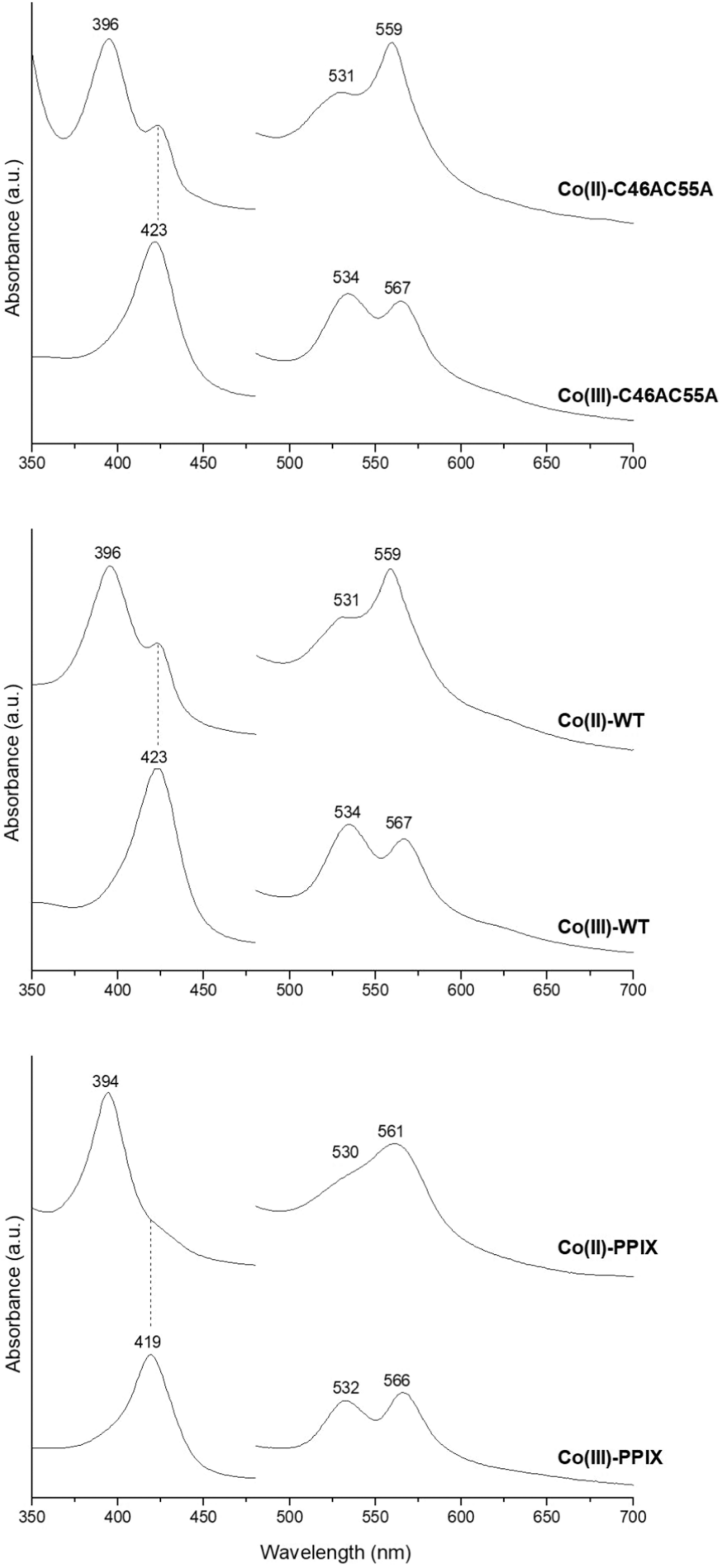
UV–vis spectra of Co(II)-PPIX, Co(III)-PPIX, Co(II)-WT, Co(III)-WT, Co(II)-C46AC55A, and Co(III)-C46AC55A at pH 7.0.

The UV–vis electronic absorption and MCD spectra of Co(II)-WT and Co(II)-C46AC55A are reported in Figures 10 and S9, respectively, together with those of Co(II)-PPIX. Degassed Co(III)-PPIX was readily reduced by addition of sodium dithionite under anaerobic conditions, although a minor amount of oxidized form is still present (Figure 10). Complete reduction was never achieved for both Co(III)-WT and Co(III)-C46AC55A, even upon the addition of a high excess of sodium dithionite anaerobically, continuous stirring, and laser irradiation. The electronic absorption (Figure 10) and MCD spectra (Figure S9) of Co(II)-WT and Co(II)-C46AC55A indicate the presence of residual Co(III) protein and some free Co(II)-PPIX, respectively.

The Soret band of Co(II)-PPIX is at 394 nm, whereas the α and β bands are observed at 561 and 530 nm, with the latter appearing as a shoulder. Its MCD spectrum is characterized by two derivative-shaped signals associated with the Soret and the Q bands and three weak peaks between 480 and 520 nm (Figure S9, Table 4). Upon binding to the Ngb scaffold, the Q bands are more resolved and the corresponding MCD derivative signal becomes sharper, moving to shorter wavelengths, similarly to other Co(II) proteins.^22,42,72,96,97^ The Soret band of the Co(II)-substituted protein derivatives is at 396 nm, and a limited sharpening of the corresponding MCD signal is observed together with a significant enhancement of the MCD signals associated with the Soret and Q bands (Figure S9). Interestingly, the electronic absorption (Figure 10) and MCD (Figure S9) spectra of Co(II)-C46AC55A are almost superimposable to those of Co(II)-WT, indicating that the small spectral differences characterizing their oxidized forms disappear upon Co(III) reduction.

**Table 4:** Wavelengths (λ, nm) of the Relevant Spectral Bands in the UV–Vis Spectra, the Second Derivative UV–Vis Spectra, and MCD Spectra of Co(II)-WT, Co(II)-C46AC55A, and Co(II)-PPIX at pH 7

	UV–vis			2nd der	MCD							
	Soret	β	α	Soret	Soret max	Soret min	Soret zerocross	β max	β min	α max	α min	α zerocross
Co(II)-WT	396	531	559	396	384	402	394	513		554	564	559
Co(II)-C46A/C55A	396	531	559	396	383	402	393	514		554	565	559
Co(II)-PPIX	394	530	561	396	387	402	394	511	523	546	573	559

**Figure 11:**
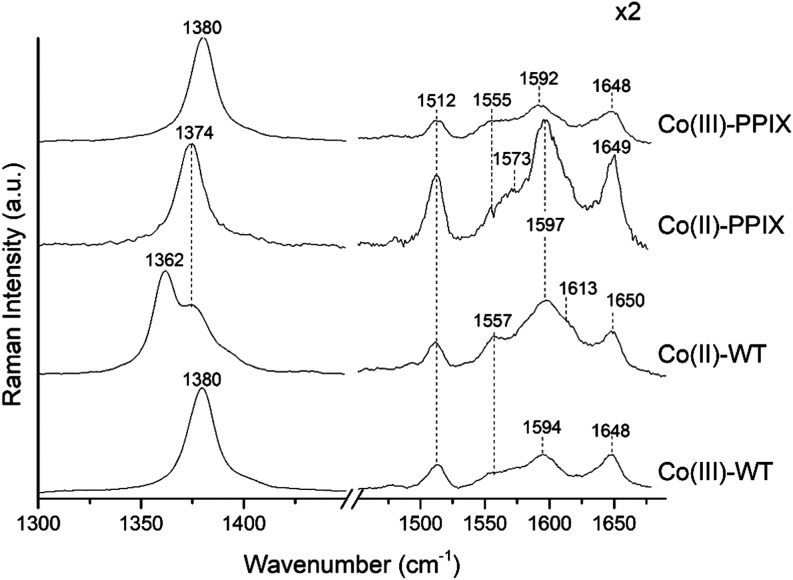
Comparison of the RR spectra in the high-wavenumber region of Co(III)-PPIX, Co(II)-PPIX, Co(III)-WT, and Co(II)-WT upon 404.8 nm excitation at pH 7.0 obtained with an 1800 grooves/mm grating.

The RR spectra of Co(II)-WT in the high-wavenumber region obtained with the 404.8 nm excitation are characteristic of a Co(II) species, as shown by the shift of the ν_4_ band from 1380 cm^–1^ (Co(III)) to lower wavenumbers (1362 cm^–1^) (Figure 11). Being the excitation wavelength in resonance with the Co(II) Soret band at 396 nm (and not with the Co(III) Soret band at 423 nm), unlike the UV–vis, no Co(III) population is detected in the RR spectrum. However, as in the MCD spectra (Figure S9), a small amount of free Co(II)-PPIX is present, as suggested by the ν_4_ band at 1374 cm^–1^. The other core size marker bands and the vinyl stretching modes wavenumbers are not significantly affected by the reduction to Co(II).

The wavenumbers of the core size marker bands are in agreement with RR studies on free Co(II) octa ethyl porphyrin in dichloromethane from ref (98) by exciting in the near-UV region at 363.8 nm. Moreover, similarities are also observed with RR data on Co(II) tetrakis porphyrins in aqueous and nonaqueous media,^99^ Co(II)-PPIX in piperidine, deoxy Co(II)-Hb and deoxy Co(II)-Mb in aqueous solution,^80^ where a laser excitation wavelength (514.5 nm) in resonance with the visible Q-band was used, resulting in the intensification of the non-totally symmetric modes. As reported in Figure S10, excitations at 404.8 and 532 nm both in nonpolarized and polarized light allowed us to assign all of the observed vibrational modes and further confirmed the presence of free Co(II)-PPIX in the protein solution upon reduction. It can be noticed that the vinyl stretching modes are almost exactly overlapped to the ν_10_ mode in both the Co(II) samples, as observed with both Soret and Q bands excitations.

Experiments in the low-wavenumber region for both Co(II)-PPIX and Co(II)-WT do not show significant differences in the bending modes of the peripheral substituents upon complexation with the protein scaffold (Figure S11).

### Effect of pH on the Spectroscopic and Redox Properties of Co-WT and Co–C46C55A

The electronic and MCD spectra for Co(III)-WT and Co(III)-C46AC55A recorded at selected pH values from 0.5 to 12.5 are shown in Figures S12–S14, and the corresponding spectral parameters at selected pH values are listed in Table S1. Below pH 7.0, the Soret and Q bands and the corresponding MCD signals of both proteins shift to higher wavelengths (Table S1) and their intensity markedly decreases (Figures S12–S14). At alkaline pH values, a more limited red shift is observed (Table S1), coupled to a smaller decrease of their intensity.

The RR spectra of Co(III)-WT recorded in the pH range from 0.75 to 12.5 do display small differences only in the vibrations of the porphyrin peripheral substituents (Figure S15). The wavenumbers of the bending modes of the propionate groups do not change as a function of pH, being at 378 and 391 cm^–1^, while their intensities show a pH dependence. Different from the upshift or the downshift of the wavenumbers, the intensity changes of these modes have not been rationalized yet.^100^ On the other hand, the two vinyl bending modes are found at 406 and 415 cm^–1^ at all investigated pH values, while one of the corresponding stretching modes in the high-wavenumber region slightly upshifts from 1648 to 1650 cm^–1^ at acid pH conditions, indicating a lower conjugation.^94^ For Co(III)-C46AC55A, similar spectral differences are observed (data not shown).

Although the 6cLS state of Co(III) appears to be maintained over the pH range investigated, the different intensities of the UV–vis absorption bands and of the corresponding MCD signals indicate that both Co(III)-WT and Co(III)-C46AC55A undergo pH-induced equilibria that influence the surroundings of the metal site.

Likewise, the far-UV CD spectra of Co(III)-WT and Co(III)-C46AC55A display remarkable changes between pH 1 and 12.5 (Figure S6), indicating that pH heavily affects their secondary structure and overall conformation. Fitting of the far-UV CD spectra with the program BeStSel^101^ indicates that between pH 7 and 4, a significant decrease of the α-helical content coupled to an increase of the amount of antiparallel β-sheets and random coil occurs. Likewise, the α-helical content significantly decreases above pH 9.5 and disappears at pH ≥ 11.8, whereas that of antiparallel β-sheets and random coil increases.

**Figure 12:**
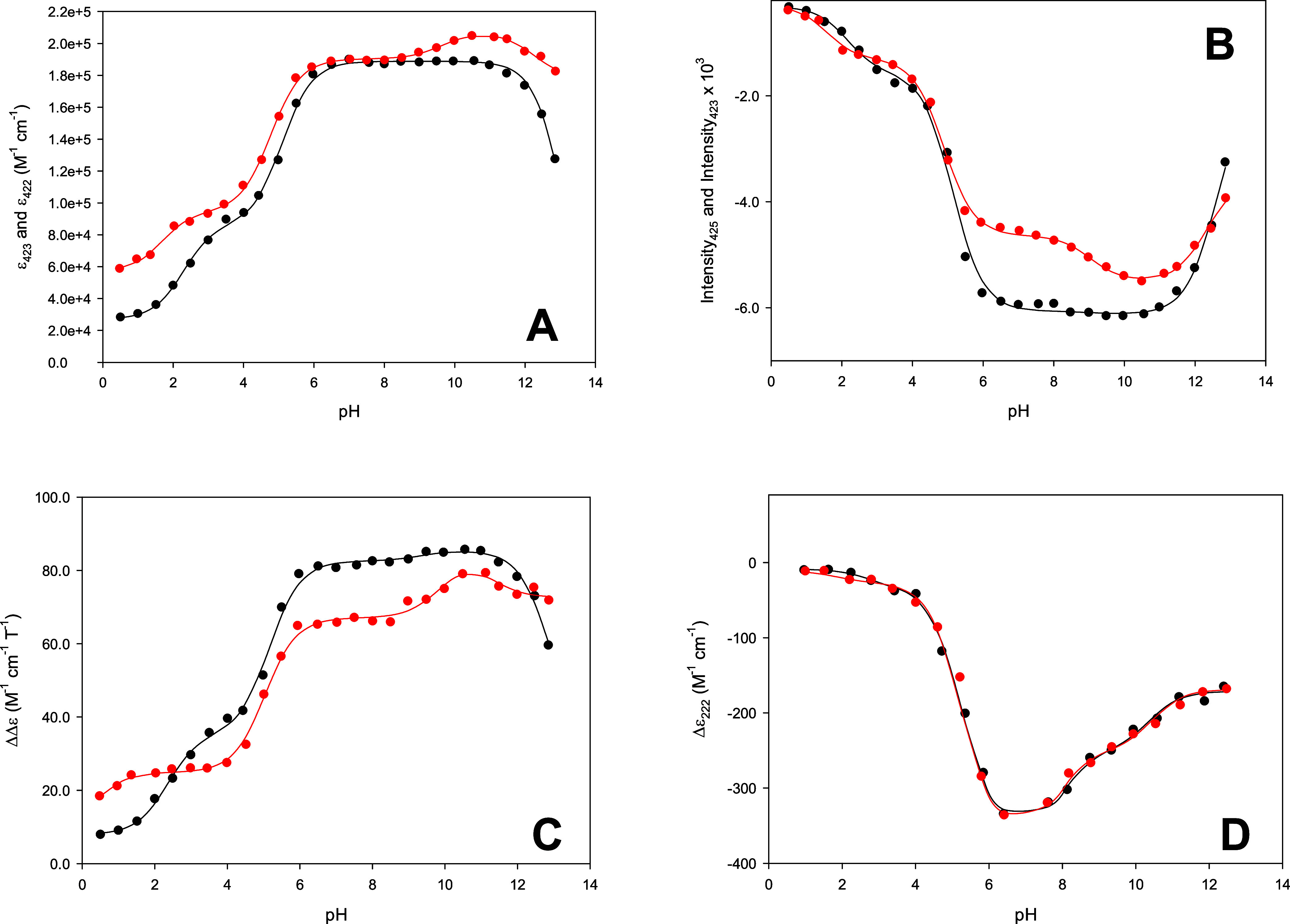
pH-induced changes in (A) the molar extinction coefficient at 423 and 422 nm (maximum of the Soret band at neutral pH), (B) intensity of the second derivative spectrum of the Soret band, (C) the peak-to-trough difference for the MCD signal associated with the Soret band, and (D) the molar ellipticity at 222 nm in Co(III)-WT (black) and Co(III)-C46AC55A (red). Solid lines are least-squares fits to eq 1 (black line A), eq 2 (red line A), eq 3 (black and red lines B), eq 4 (black and red lines C), and eq 5 (black and red lines D), respectively.

The changes in absorbance of the Soret band and in the intensity of its second derivative, in the peak-to-trough distance of the MCD Soret signal, and in the difference between the extinction coefficient for the left- and right-hand circular polarized light at 222 nm at different pH values for the Co(III)-WT and Co(III)-C46AC55A are reported in Figure 12 and were interpolated using eqs 1, 2, 3, 4, and 5 reported in the Materials section.^34^

It turns out that in the pH range investigated, the electronic properties of the metal center and the protein secondary structure of Co(III)-substituted WT and C46C55A are influenced by five acid–base equilibria, as observed for the corresponding ferric proteins.^34^ The apparent pK_a_ values obtained for Co(III)-WT and Co(III)-C46AC55A from the interpolation process are reported in Table 5.

**Table 5:** Apparent pK_a_ Values of the Acid/base Equilibria Influencing the Electronic Properties of the Metal Center and the Secondary Structure of Co(III)-WT and Co(III)-C46AC55A

Co(III)-WT					Co(III)-C46AC55A				
	UV–vis	2nd der	MCD	Far-UV CD		UV–vis	2nd der	MCD	Far-UV CD
pK_a1_^a^	2.3	2.2	2.3	2.7	pK_a1_	1.7	1.6	0.4	1.8
pK_a2_^a^	5.1	5.1	5.2	4.6	pK_a2_	4.8	4.9	5.0	5.2
pK_a3_^a^				8.1	pK_a3_				8.0
pK_a4_^a^		9.0	9.3	10.4	pK_a3_	9.6	9.0	9.8	10.5
pK_a5_^a^	13.2	12.6			pK_a5_	12.3	12.3	11.4	

a associated error ± 0.2.

To the best of our knowledge, the Co(III) adduct of microperoxidase-8 (MP8, a proteolytic fragment of cytochrome *c*, containing the heme group and 8 amino acids^69^) is the only Co-substituted heme protein whose spectroscopic properties were analyzed in detail between pH 1 and 13 so far.^69^ At pH 7, Co(III)-MP8 contains a low-spin six-coordinated Co(III), whose axial coordination positions are occupied by the imidazolic nitrogen of the proximal histidine and a H_2_O molecule.^69^ Between pH 0.5 and 13.0, the electronic properties of the metal center of Co(III)-MP8 are influenced by five acid–base equilibria, involving the metal axial ligands and the propionate groups of Co(III)-PPIX.^69^

The electronic properties of the metal center of Co(III)-WT and Co(III)-C46AC55A and their *E*°′_Co(III)/Co(II)_ values (see below) are significantly influenced by two acid–base equilibria occurring below pH 7, corresponding to pK_a1_ and pK_a2_, which do not alter the likely bis-His axial coordination and low-spin state of Co(III). The changes in the spectroscopic properties observed below pH 4 for both Co(III)-WT and Co(III)-C46AC55A (pK_a1_) are similar to those reported in the same pH interval for Co(III)-MP8,^69^ which were attributed to the release of Co(III)-PPIX by the protein scaffold,^69^ although in the present case free Co(III)-PPIX was not observed in the RR spectra.

The acid–base equilibrium occurring under slightly acidic conditions, corresponding to pK_a2_, induces relevant changes in the electronic, MCD, and far-UV CD spectra of Co(III)-WT and Co(III)-C46AC55A. Hence, it influences the electronic properties of the protein-bound Co(III)-PPIX and heavily modifies the secondary structure of both proteins. Moreover, it apparently affects the propionate groups, whose bending mode intensities slightly change in the same pH interval (Figure S15). In analogy with Co(III)-MP8^69^ and Fe(III)-WT and Fe(III)-C46AC55A,^34^ it is possible that the acid–base equilibrium corresponding to pK_a2_ arises from one (or both) of the heme propionates of the protein-bound Co(III)-PPIX. This is consistent with the significant protein unfolding observed, since the carboxylate groups of both propionates are involved in a network of noncovalent interactions crucial for the protein structure.^25,26,31,33^ Fe(III) replacement with Co(III) induces a limited decrease in the observed pK_a2_ values, in agreement with the increased electron density of Co(III)-PPIX compared to the ferric heme. Moreover, as in the case of the ferric proteins,^34^ the pK_a2_ values of Co(III)-WT and Co(III)-C46AC55A are nearly identical, indicating that the corresponding acid–base equilibrium is not significantly influenced by the absence of the Cys46/Cys55 disulfide bridge.

Above neutrality, three acid–base equilibria are observed (Table 5). The one corresponding to pK_a3_ slightly lowers the α-helix content of both proteins without affecting the metal center. Therefore, it should involve a residue located outside the protein cleft surrounding the metal center, which is not significantly affected by the presence of the Cys46/Cys55 disulfide bond, being the pK_a3_ of Co(III)-WT and Co(III)-C46AC55A almost coincident (Table 5). This acid–base equilibrium observed for the Fe(III)-WT and Fe(III)-C46AC55A was tentatively attributed to the imidazole ring of His23, which belongs to helix B and is located on the protein surface at more than 17 Å from the metal,^25,26,34^ although a contribution by a pH-dependent variation of populations of the two different conformers, containing a reversed and a canonical heme insertion orientation, cannot be excluded.

The acid–base equilibrium occurring between pH 8.5 and 11.0, associated with pK_a4_, exerts a limited influence on the spectroscopic properties of the metal center, in particular for Co(III)-WT, whereas it heavily impacts on the secondary structure of both Co(III)-substituted proteins, as indicated by the almost complete disappearance of the α-helices. Moreover, it significantly influences the reduction potential of the Co(III)/Co(II) couple in both Co(III)-WT and Co(III)-C46AC55A (pK_aox3_, Table 6). As proposed for the ferric proteins, the equilibrium possibly involves the solvent-exposed ε-amino group of Lys67,^34^ whose pK_a_ could be lowered due to its involvement as an H-bond donor in the distal H-bonding network.^23,25,26,33−36,102−106^ The smaller effect that this reaction exerts on the electronic properties of the metal center of Co(III)-WT compared to Co(III)-C46AC55A is quite surprising, since in the ferric proteins, the opposite effect is observed.^34^

**Table 6:** Apparent pKa Values of the Acid/base Equilibria Influencing the *E*°′ of the Co(III)/Co(II) Couple of Co-WT and Co–C46AC55A^a^

Co-WT				Co–C46AC55A			
pK_aox1_^b^	1.9	pK_ared1_^b^	4.3	pK_aox1_^b^	2.0	pK_ared1_^b^	3.1
pK_aox2_^b^	4.9	pK_ared2_^b^	6.3	pK_aox2_^b^	4.7	pK_ared2_^b^	6.2
pK_aox3_^b^	10.0	pK_ared3_^b^	11.6	pK_aox3_^b^	9.9	pK_ared3_^b^	10.8

a Base electrolyte 5 mM phosphate buffer plus 50 mM KClO_4_, *T* = 20 °C.

b associated error ± 0.2.

The acid–base equilibria observed at higher pH values most probably arise from different phenomena. For Co(III)-WT, the acid–base equilibrium with pK_a5_ around 13 seems to be associated with the pH-induced release of Co(III)-PPIX as a consequence of protein unfolding, indicated by the dramatic changes observed in the far-UV CD spectra, although the RR spectra did not evidence any free Co(III)-PPIX. The different spectroscopic changes in the UV–vis and MCD spectra observed for Co(III)-C46AC55A up to pH 12 suggest that they arise from an acid–base equilibrium not observed for Co(III)-WT and that the release of Co(III)-PPIX shifts at even higher pH values, falling above the pH interval analyzed. Therefore, it appears that deletion of the Cys46/Cys55 disulfide bridge somehow stabilizes the protein environment surrounding the metal center under strongly basic conditions.

**Figure 13:**
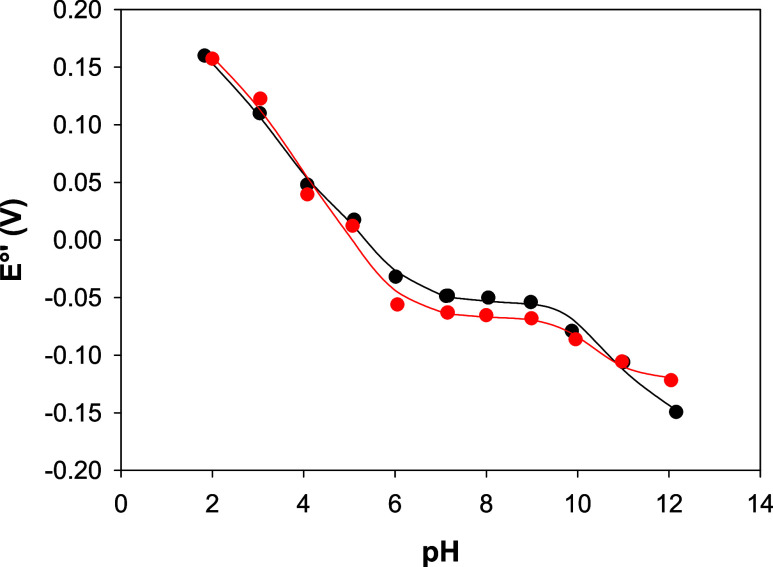
*E*°′_Co(III)/Co(II)_ vs pH profiles for Co-WT (black) and Co–C46AC55A (red). Solid lines are least-squares fits to eq 6. Base electrolyte 5 mM phosphate buffer plus 50 mM KClO_4_, *T* = 20 °C.

The reduction potentials of the Co(III)/Co(II) couple in both Co-WT and Co–C46AC55A markedly decrease upon changing the pH from 2 to 12.5 (Figure 13), showing that in the pH interval considered, Co(III) reduction is coupled with one or more protonation processes.

The *E*°′ _Co(III)/Co(II)_ vs pH profiles of Co-WT and Co–C46AC55A immobilized on PGE were fitted using a three-equilibrium Clark Dutton equation^34,54,55^ (eq 6), indicating that in the pH range investigated, the redox properties of the metal center of both Co-WT and Co–C46AC55A are influenced by at least three acid–base equilibria, whose apparent pK_a_ values in the oxidized and reduced forms are reported in Table 6.

The pK_aox_ values reported in Table 6 are comparable to the average pK_a1_, pK_a2_, and pK_a4_ values reported in Table 5 for the Co(III) species, suggesting that the redox properties of both Co-WT and Co–C46AC55A are affected by the same acid–base equilibria influencing their spectroscopic and structural properties. On the other hand, no straightforward relationship exists between the above pH-induced conformational equilibria and the pH dependence of the electrocatalytic parameters for H_2_ production, as the former are relative to the Co^3+^ species and the Co^3+^/Co^2+^ redox couple, whereas the electrocatalytic production of H_2_ most probably involves the Co^1+^/Co° redox couple.^12,13,18,19^

## Conclusions

This paper presents a comprehensive electrochemical and spectroscopic characterization of the Co-substituted derivatives of WT human neuroglobin and its C46A/C55A mutant, demonstrating the ability of Co-WT and Co–C46AC55A to mediate the electrocatalytic reduction of water protons to H_2_ as well as providing new information on the influence of metal axial ligation and the protein matrix on the electronic properties and redox reactivity of protein-embedded Co-PPIX.

The Co(III) and Co(II) derivatives of WT and C46C55A invariably contain a low-spin six-coordinated Co, whose electronic properties are scarcely sensitive to the deletion of the Cys46-Cys55 disulfide bond and to the nature of axial ligands. The similarities between their UV–vis and MCD spectra and those of the adducts of Co(III)-Mb with nitrogenous axial ligands as well as the reduction thermodynamics of the Co(III)/Co(II) redox couple are both consistent with bis-His axial coordination. Co insertion prevents the heme rotation disorder observed in ferric proteins. Moreover, the overall pH-dependent behavior and the overall mechanism modulating the *E*°′_Co(III)/Co(II)_ appears to be not significantly affected by the replacement of the heme *b* with Co-PPIX, suggesting that the same molecular factors are operative in both cases and that the observed differences mainly arise from the different chemical properties of Fe and Co. Upon physiadsorption on PGE, both Co-WT and Co–C46AC55A catalyze the reduction of H_3_O^+^ to H_2_, featuring onset potentials and overpotentials comparable to those of Co-porphyrin/polypeptide catalysts. On the other hand, the postulated His-His six coordination exerts a double-edged effect on the electrocatalytic ability for the development of H_2_ of Co-WT and Co–C46AC55A, slightly lowering their electrocatalytic efficiency compared to six-coordinated aquo-His Co-Mb but making it rather insensitive to the presence of O_2_.

## Supporting Information Available

Plot of *E*°′ Co(III)/Co(II) vs temperature for Co-WT and Co–C46C55A; cyclic voltammetries of Co-PPIX and Co-WT on PGE at pH 6, 7, and 8; pH-induced changes of the onset potential of the voltammetric signal associated with the electrocatalytic reduction of H_3_O^+^ to H_2_ for Co-WT and Co–C46C55A; pH-induced changes of the catalytic current density (at −1.12 V) of the voltammetric signal associated with the electrocatalytic reduction of H_3_O^+^ to H_2_ for Co-WT and Co–C46C55A; temperature-induced changes of the catalytic current density and the onset potential of the voltammetric signal associated with the electrocatalytic reduction of H_3_O^+^ to H_2_ for Co-WT and Co–C46C55A; far-UV CD spectra of Co(III)-WT and Co(III)-C46AC55A at different pH values; RR spectra of Co(III)-PPIX and Co(III)-WT taken in polarized light obtained with the 404.8, 441.6, and 532 nm laser excitations at pH 7.0; curve-fitting analysis of the RR spectrum of Co(III)-WT obtained with the 441.6 nm laser excitation at pH 7.0; MCD spectra of Co(II)-WT, Co(II)-C46AC55A, and Co(II)-PPIX at pH 7.0; RR spectra in the high-wavenumber region of Co(II)-PPIX and Co(II)-WT upon 404.8 and 532 nm excitation at pH 7.0, taken in polarized light with a 3600 or 1800 grooves/mm grating; RR spectra in the low-wavenumber region of Co(II)-PPIX and Co(II)-WT upon 404.8 nm excitation at pH 7.0; electronic absorption spectra in the visible region of Co(III)-WT and Co(III)-C46AC55A at different pH values; 2nd derivative electronic absorption spectra in the visible region of Co(III)-WT and Co(III)-C46AC55A at different pH values; MCD spectra in the visible region of Co(III)-WT and Co(III)-C46AC55A at different pH values; RR spectra of Co(III)-WT at different pH values; wavelengths of the relevant spectral bands in the UV–vis spectra; and second derivative UV–vis spectra and MCD spectra of Co(III)-WT and Co(III)-C46AC55A at different pH values (PDF)

## Abbreviations

hNgb: human neuroglobin
Fe(III)-WT: WT ferric human neuroglobin
Fe(III)-C46AC55A: ferric human neuroglobin C46AC55A mutant
Co(III)/Co(II)-WT: Co(III)- or Co(II)-substituted WT human neuroglobin
Co(III)/Co(II)-C46AC55A: Co(III)- or Co(II)-substituted human neuroglobin C46AC55A mutant
Co(III)/Co(II)-PPIX: Co(III) or Co(II) protoporphyrin IX
Mb: myoglobin
Co(III)/Co(II)-Mb: Co(III)/Co(II)-substituted myoglobin
Hb: hemoglobin
*CV*: cyclic voltammetry
MCD: magnetic circular dichroism
RR: resonance Raman
6cLS: six-coordinated low spin
