# Supplementary material for: Electrochemical and Spectroscopic Characterization of Co-Neuroglobin: A Bioelectrocatalyst for H2 Production
Source: Inorg Chem. 2025 May 2;64(18):9066–83. doi: 10.1021/acs.inorgchem.5c00551 (PMC12076554; doi:10.1021/acs.inorgchem.5c00551)
Supplement: Supplementary file 1 — ic5c00551_si_001.pdf [file ic5c00551_si_001.pdf]

# ELECTROCHEMICAL AND SPECTROSCOPIC CHARACTERIZATION OF Co-NEUROGLOBIN: A BIOELECTROCATALYST FOR $H_2$ PRODUCTION

*Mirco Meglioli[§], Federico Sebastiani[#], Marzia Bellei[‡], Giulia Di Rocco[‡], Antonio Ranieri[‡], Carlo Augusto Bortolotti[‡], Marco Sola[‡], Marco Borsari[§], Giulietta Smulevich[#], Gianantonio Battistuzzi[§*].*

[§] *Department of Chemical and Geological Sciences, University of Modena and Reggio Emilia, via Campi 103, 41125 Modena, Italy*

[#] *Department of Chemistry "Ugo Schiff" DICUS, University of Florence, via della Lastruccia 3, 50019 Sesto Fiorentino (FI), Italy*

[‡] *Department of Life Sciences, University of Modena and Reggio Emilia, via Campi 103, 41125 Modena, Italy*

*Corresponding authors: Phone: +39-0592058642; e-mail: gianantonio.battistuzzi@unimore.it

# Supplementary Information

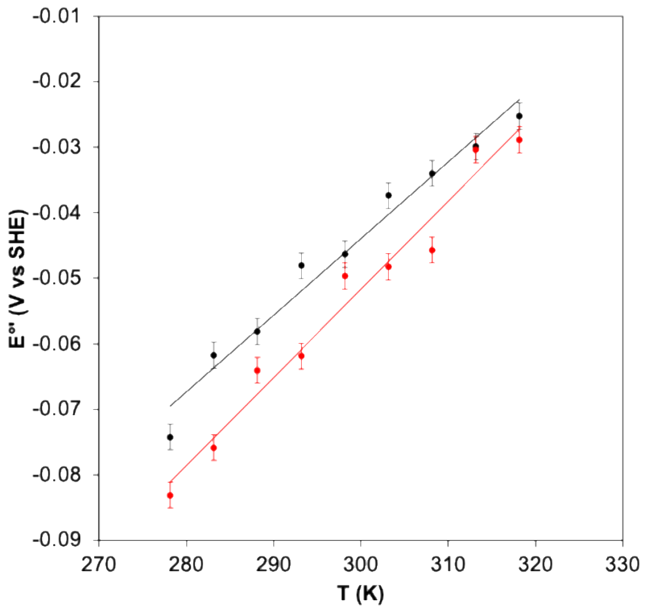


**Figure S1**: Plot of E°' $_{Co(III)/Co(II)}$ vs. temperature for Co-WT (black) and Co-C46C55A (red). Base electrolyte 5 mM phosphate buffer plus 50 mM $KClO_4$, pH 7.0.

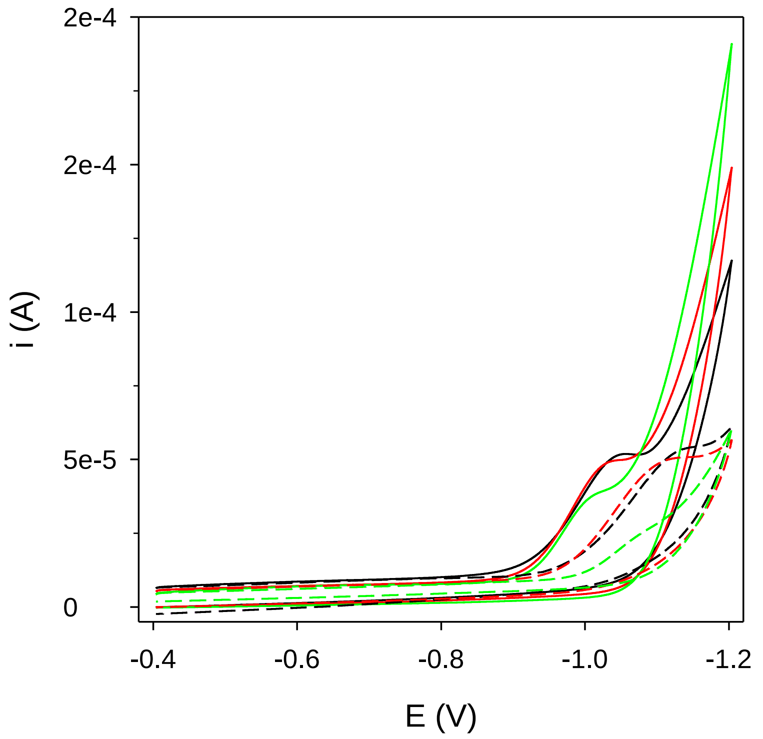


**Figure S2**: Cyclic voltammetry of 3 μM Co-PPIX (solid line) and Co-WT (dashed line) on PGE, at pH 6 (black), 7 (red) and 8 (green), Ar atmosphere. T=20°C, base electrolyte 5 mM tris-HCl buffer plus 50 mM NaCl.

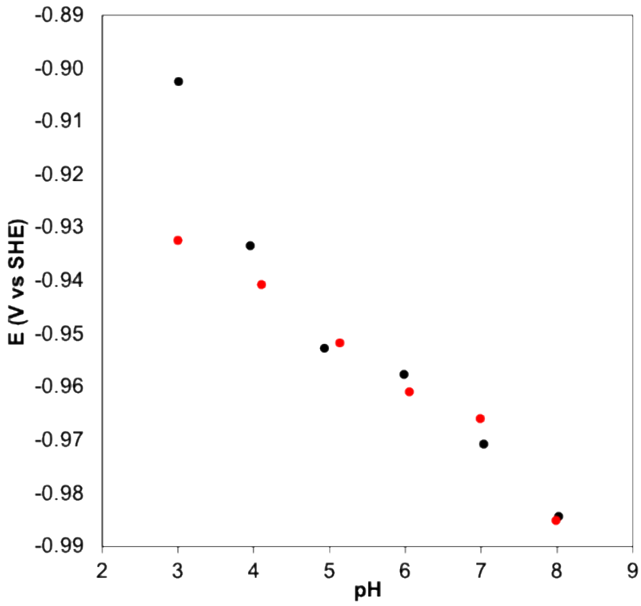


**Figure S3**: Influence of pH on the onset potential of the electrocatalytic wave for reduction of $H_3O^+$ to $H_2$ for Co-WT (black) and Co-C46AC55A (red). Base electrolyte 5 mM tris-HCl buffer plus 50 mM NaCl, T=20°C, Ar atmosphere.

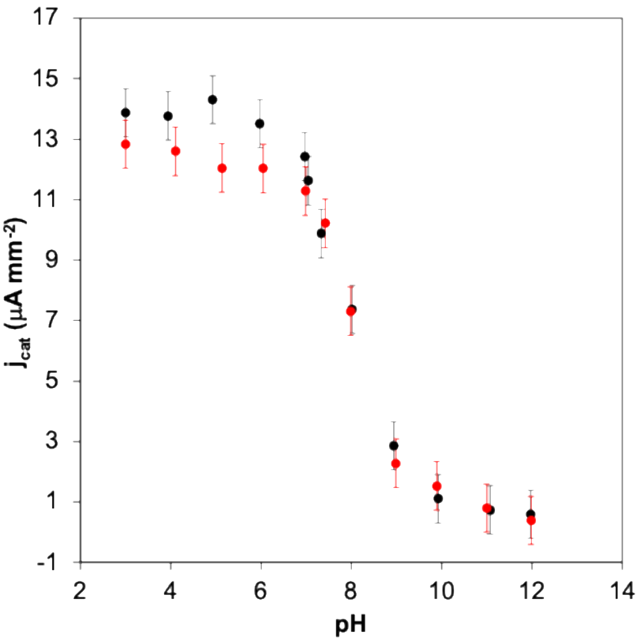


**Figure S4**: Influence of pH on the electrocatalytic current density ($j_{cat}$) for $H_2$ evolution measured at -1.12 V for Co-WT (black) and Co-C46AC55A (red). Base electrolyte 5 mM tris-HCl buffer plus 50 mM NaCl, T=20°C, Ar atmosphere.

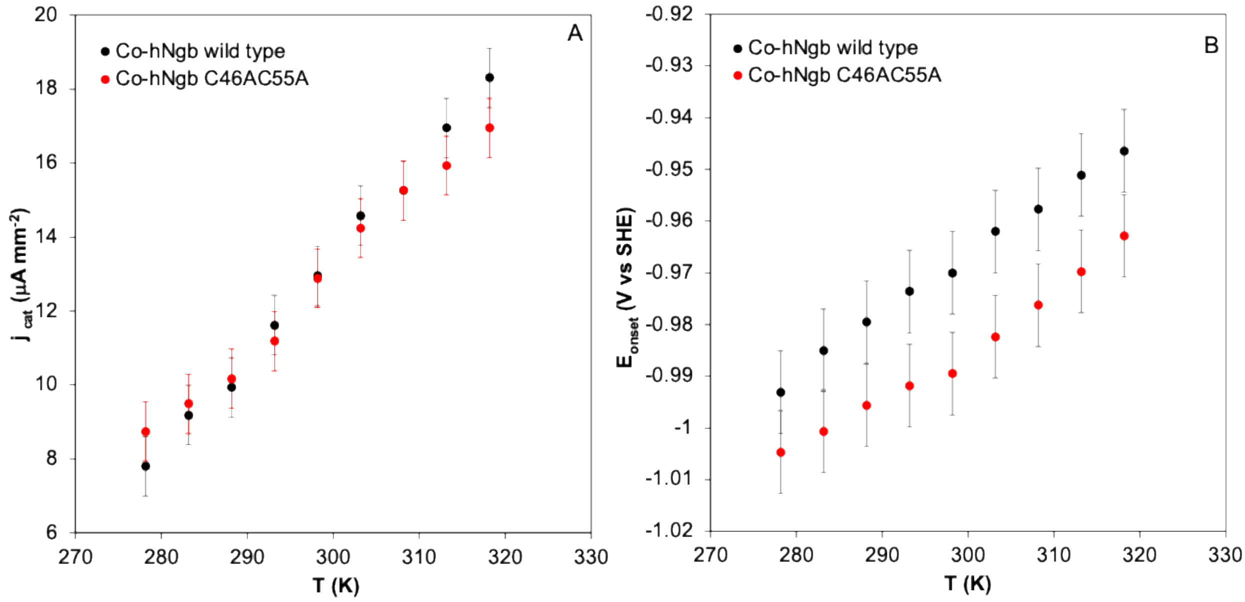


**Figure S5**: Temperature-induced changes of (A) the catalytic current density ($j_{cat}$) and (B) the onset potential ($E_{onset}$) of the voltammetric signal associated to the electrocatalytic reduction of $H_3O^+$ to $H_2$ for Co-WT (black) and Co-C46C55A (red). Base electrolyte 5 mM tris-HCl buffer plus 50 mM NaCl, pH 7.0, Ar atmosphere.

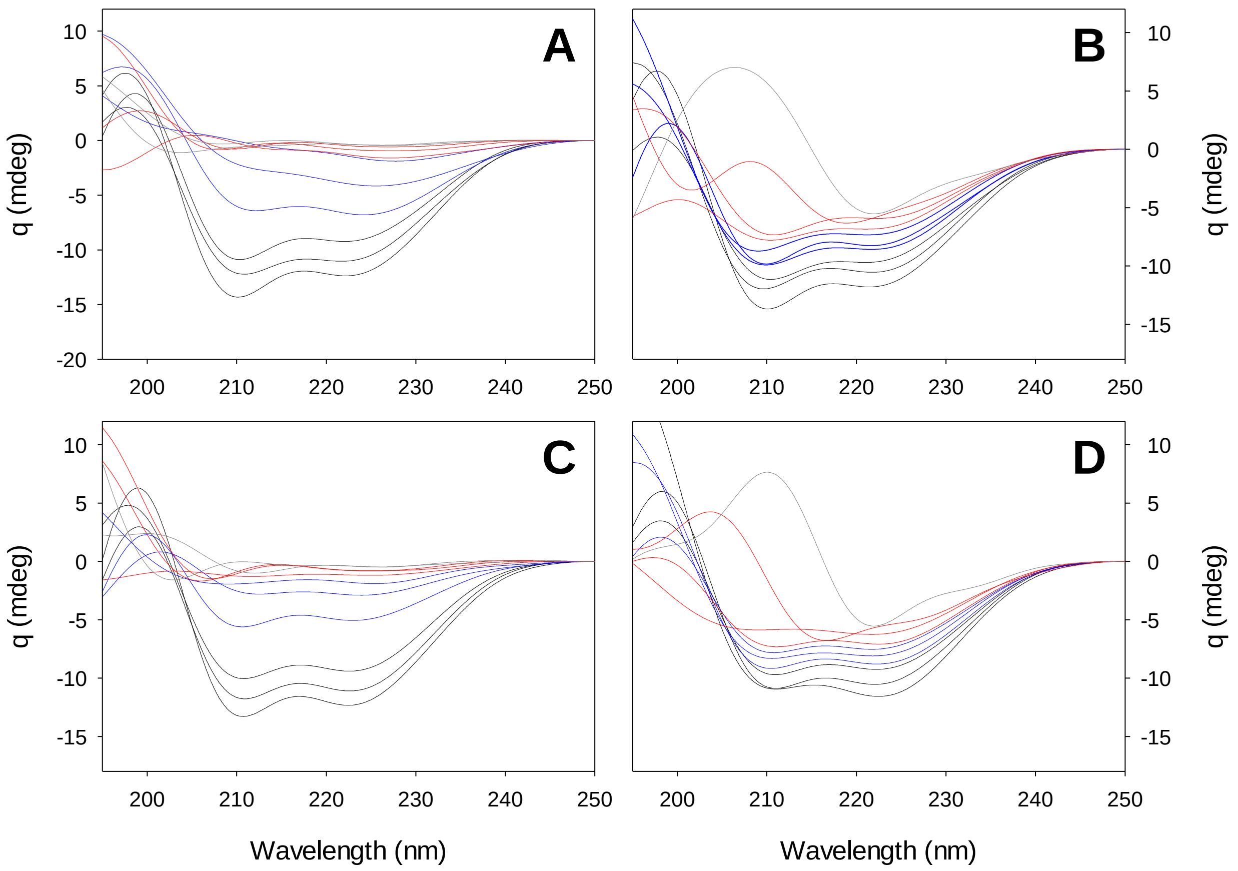


**Figure S6**: Far UV CD spectra of Co(III)-WT and Co(III)-C46AC55A at selected acidic (A, C) and basic (B, D) pH values, in 5 mM phosphate buffer plus 0.01 M NaCl. Acid pH values: 5.5 < pH < 7.0, black, 3.5 < pH < 5.5, blue; 2.0 < pH < 3.5, red; 1.0 < pH < 2.0, dark gray. Basic pH values: 7.0 < pH < 8.5, black, 8.5 < pH < 10.0, blue; 11 < pH < 12.0, red; pH = 12.4, dark gray. Protein concentration is 1 μM.

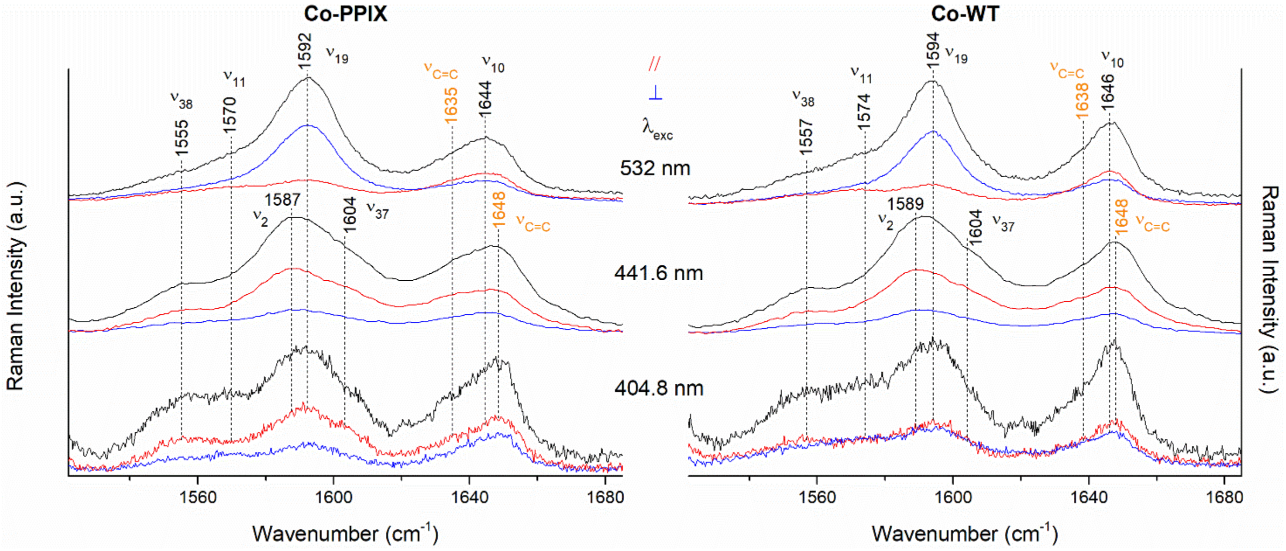


**Figure S7** Comparison of the RR spectra of Co(III)-PPIX (Co-PPIX) and Co(III)-WT (Co-WT) obtained with the 404.8 nm, 441.6 nm, and 532 nm laser excitations at pH 7.0, taken in polarized light (parallel, in red, and perpendicular, in blue). The vinyl stretching modes are reported in orange.

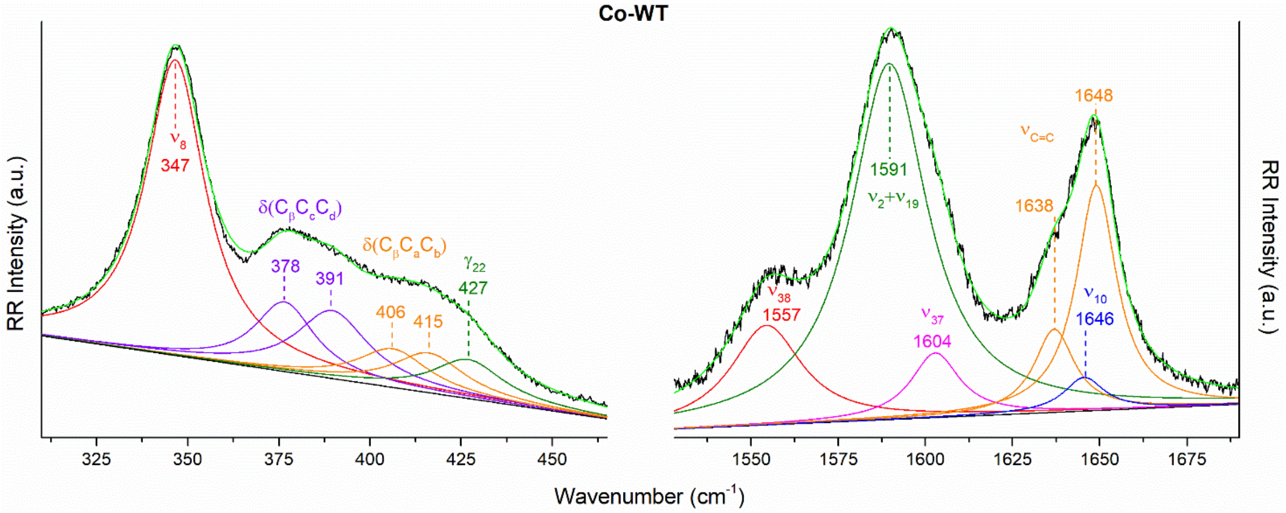


**Figure S8** Curve-fitting analysis of the RR spectrum of Co(III)-WT (Co-WT) obtained with the 441.6 nm laser excitation at pH 7.0. In the low wavenumber region (left panel) the bandwidths are 20, 20, 24, 24, 24 and 24 $cm^{-1}$ for the $\nu_8$, two $\delta(C_\beta C_c C_d)$, two $\delta(C_\beta C_a C_b)$ and $\gamma_{22}$ modes, respectively. In the high wavenumber region (right panel) the bandwidths are 25, 27, 17, 14 and 14 $cm^{-1}$ for the $\nu_{38}$, $\nu_2 + \nu_{19}$, $\nu_{37}$ , $\nu_{10}$ and two $\nu_{C=C}$ modes, respectively. Propionate and vinyl vibrations are reported in purple and orange, respectively. The modes are assigned according to S. Hu, K.M. Smith, T.G. Spiro, J. Am. Chem. Soc. 1996, 118, 12638-12646.

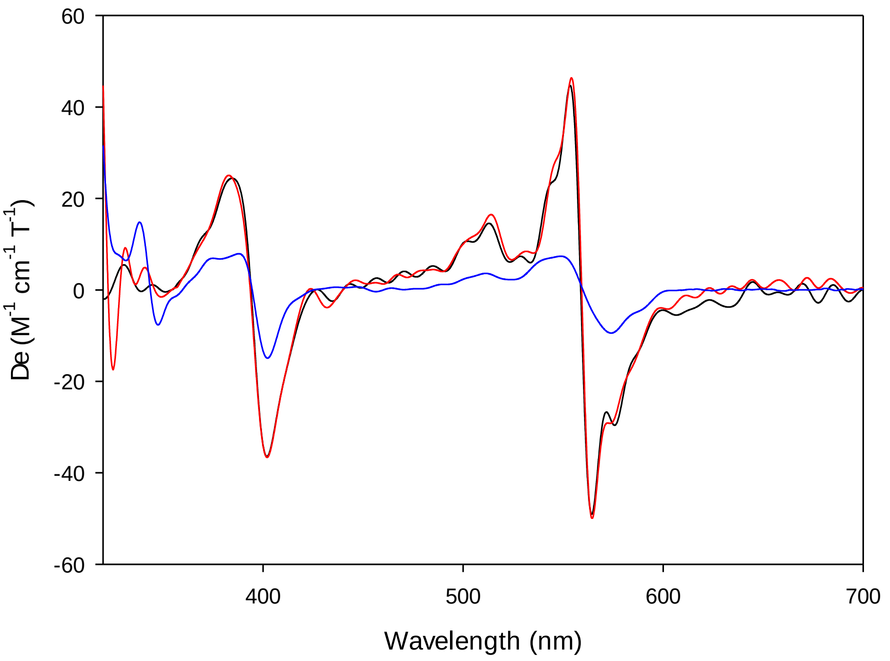


**Figure S9**. MCD spectra of Co(II)-WT (black), Co(II)-C46AC55A (red) and Co(II)-PPIX (blue) at pH 7.0. Protein and Co-PPIX concentrations are 10 μM and 20 μM, respectively.

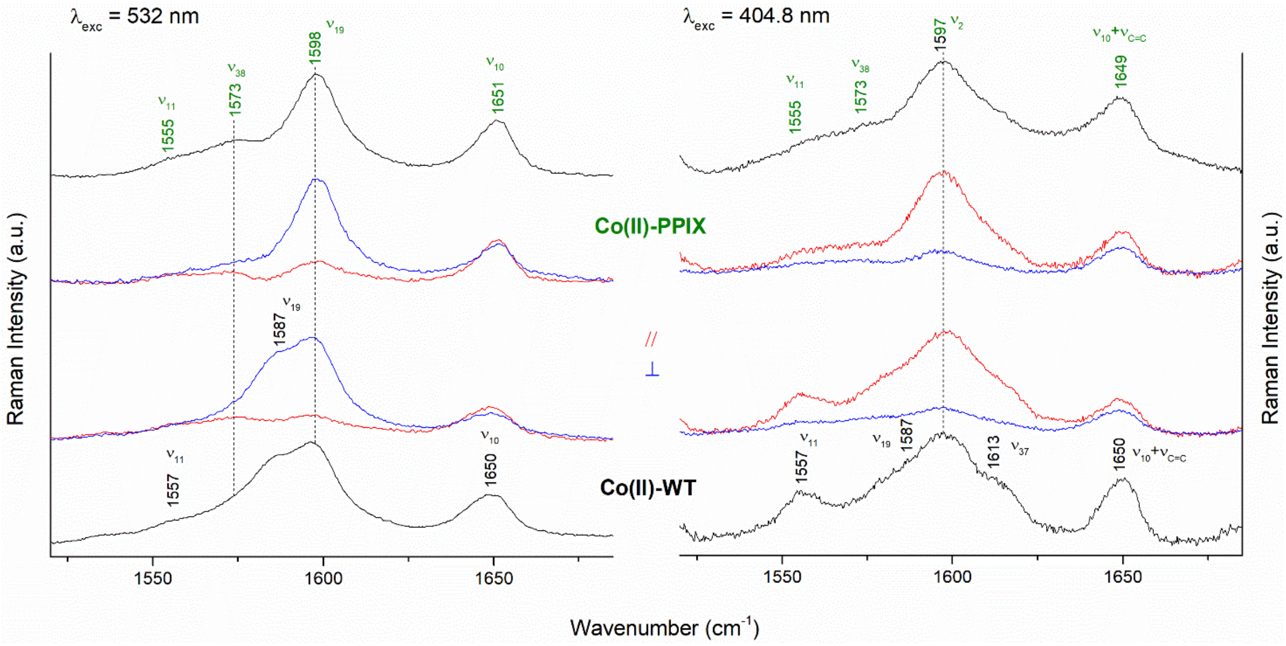


**Figure S10**. RR spectra in the high wavenumber region of Co(II)-PPIX (top) and Co(II)-WT (bottom) upon 404.8 nm (right) and 532 nm (left) excitation at pH 7.0, with a 3600 or 1800 grooves/mm grating, respectively. The spectra taken in polarized light are also reported in red (parallel) and blue (perpendicular), while the wavenumbers of the free Co(II)-PPIX and Co(II)-WT bands are indicated in green and black, respectively. The spectra have been normalized to the most intense band in the selected wavenumber range.

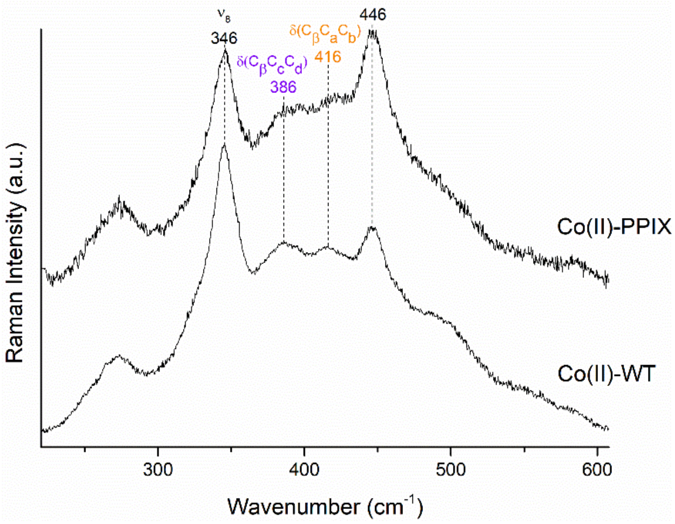


**Figure S11.** RR spectra in the low wavenumber region of Co(II)-PPIX and Co(II)-WT upon 404.8 nm excitation at pH 7.0. Propionate and vinyl bending modes are reported in purple and orange, respectively.

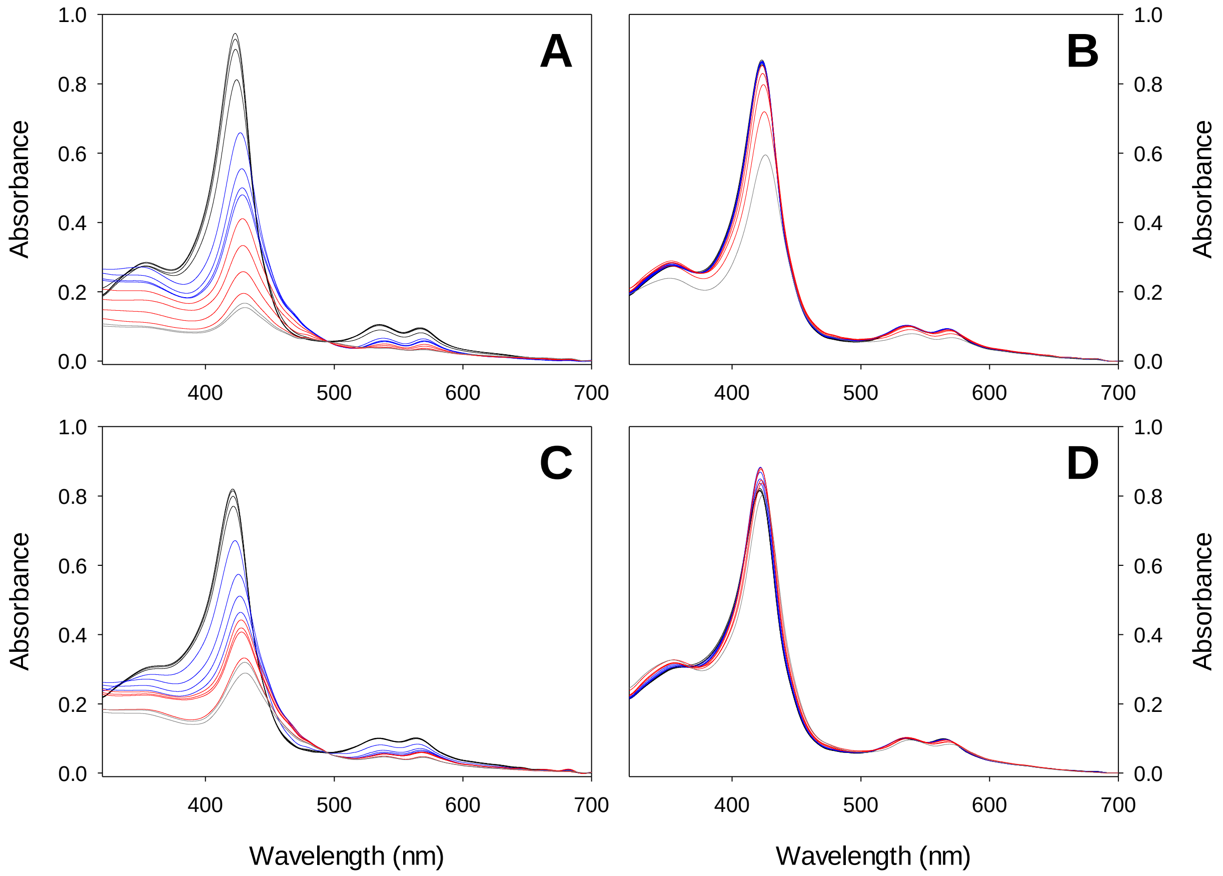


**Figure S12**: Electronic absorption spectra in the visible region of Co(III)-WT and Co(III)-C46AC55A at selected acidic (A, C) and basic (B, D) pH values, in 50 mM phosphate buffer plus 0.1 M NaCl. Acid pH values: 5.5 < pH < 7.0, black, 3.5 < pH < 5.0, blue; 1.5 < pH < 3.0, red; 0.5 < pH < 1.0, dark gray. Basic pH values: 7.0 < pH < 8.5, black, 9.0 < pH < 10.5, blue; 11 < pH < 12.5, red; pH = 13.0, dark gray. Protein concentration is 10 μM.

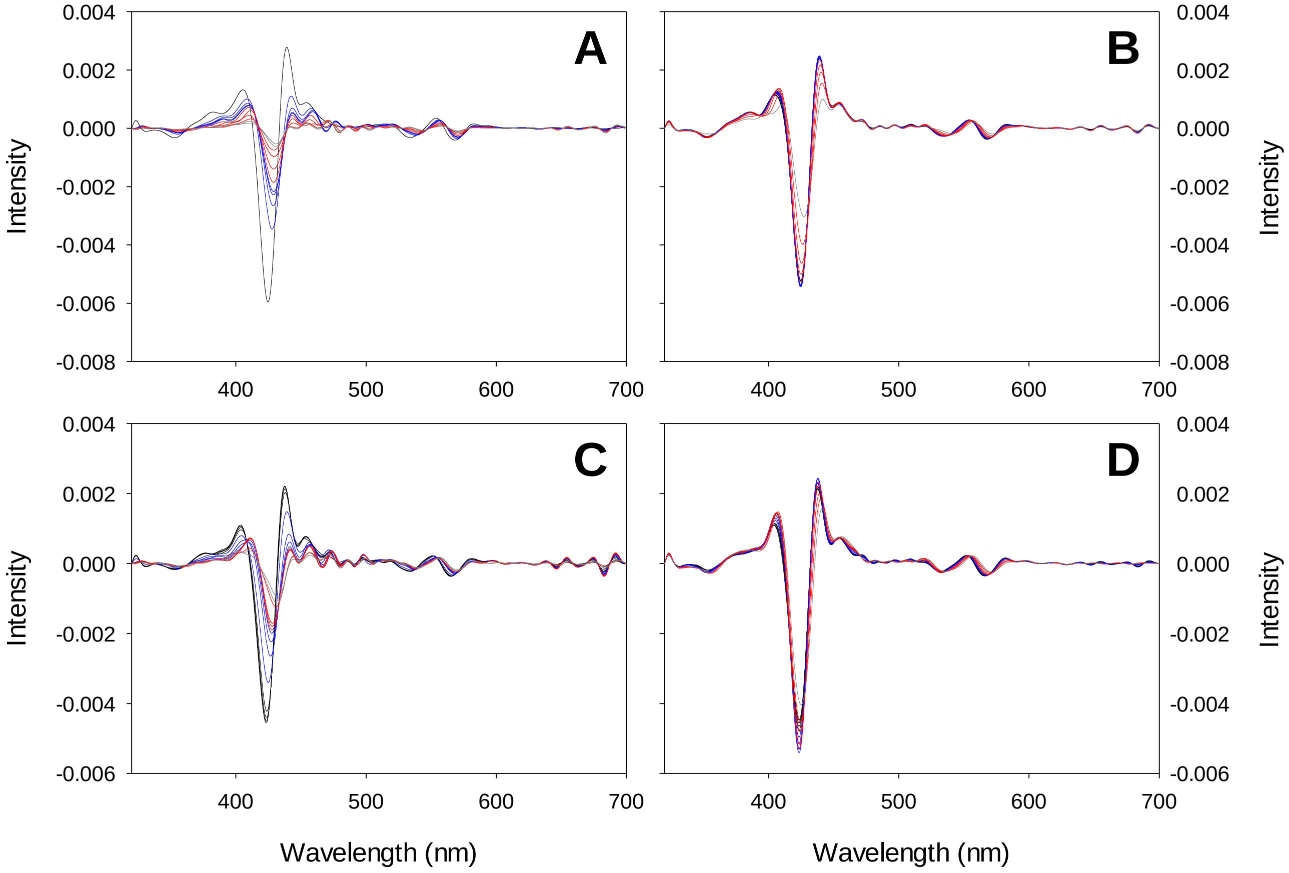


**Figure S13**: 2nd derivative electronic absorption spectra in the visible region of Co(III)-WT and Co(III)-C46AC55A at selected acidic (A, C) and basic (B, D) pH values, in 50 mM phosphate buffer plus 0.1 M NaCl. Acid pH values: 5.5 < pH < 7.0, black, 3.5 < pH < 5.0, blue; 1.5 < pH < 3.0, red; 0.5 < pH < 1.0, dark gray. Basic pH values: 7.0 < pH < 8.5, black, 9.0 < pH < 10.5, blue; 11 < pH < 12.5, red; pH = 13.0, dark gray. Protein concentration is 10 μM.

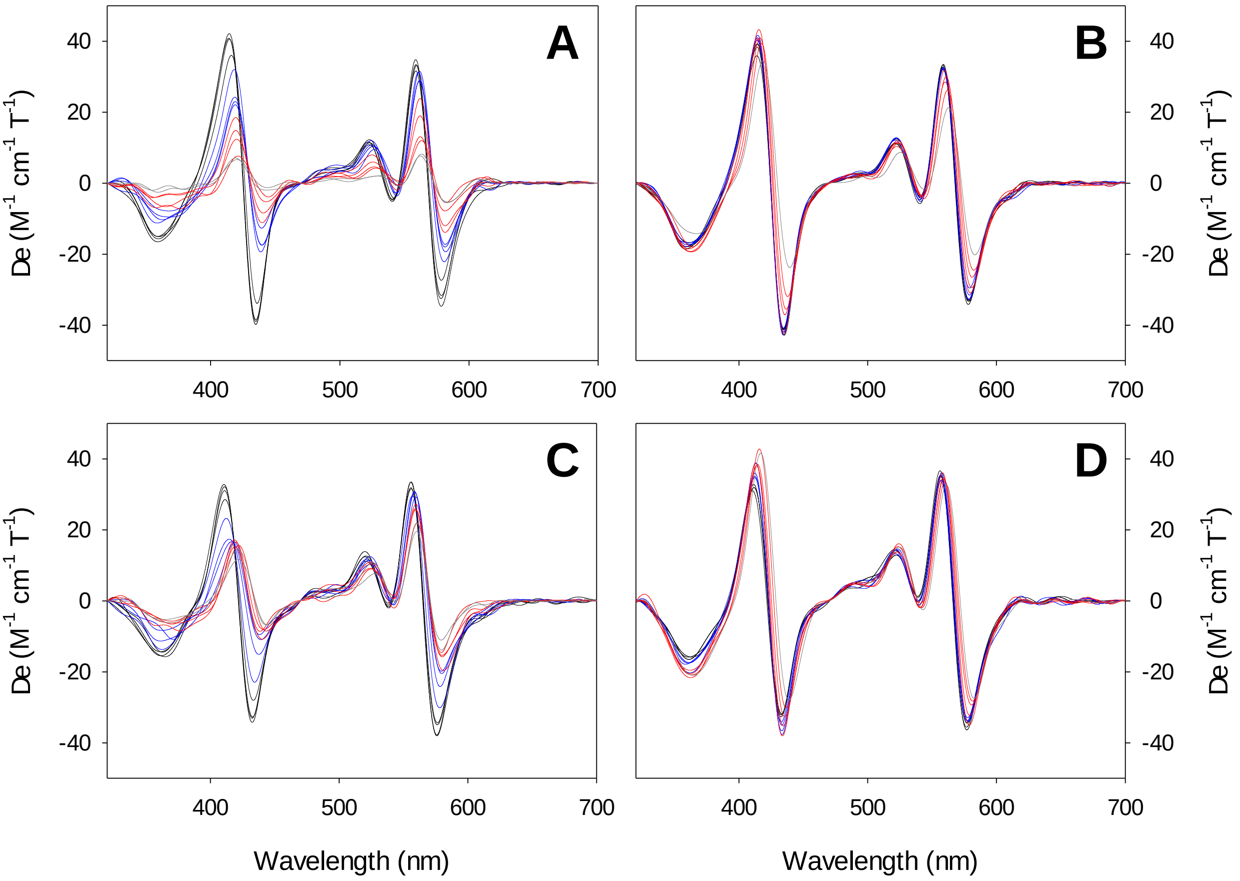


**Figure S14**: MCD spectra in the visible region of Co(III)-WT and Co(III)-C46AC55A at selected acidic (A, C) and basic (B, D) pH values, in 50 mM phosphate buffer plus 0.1 M NaCl. Acid pH values: 5.5 < pH < 7.0, black, 3.5 < pH < 5.0, blue; 1.5 < pH < 3.0, red; 0.5 < pH < 1.0, dark gray. Basic pH values: 7.0 < pH < 8.5, black, 9.0 < pH < 10.5, blue; 11 < pH < 12.5, red; pH = 13.0, dark gray. Protein concentration is 10 μM.

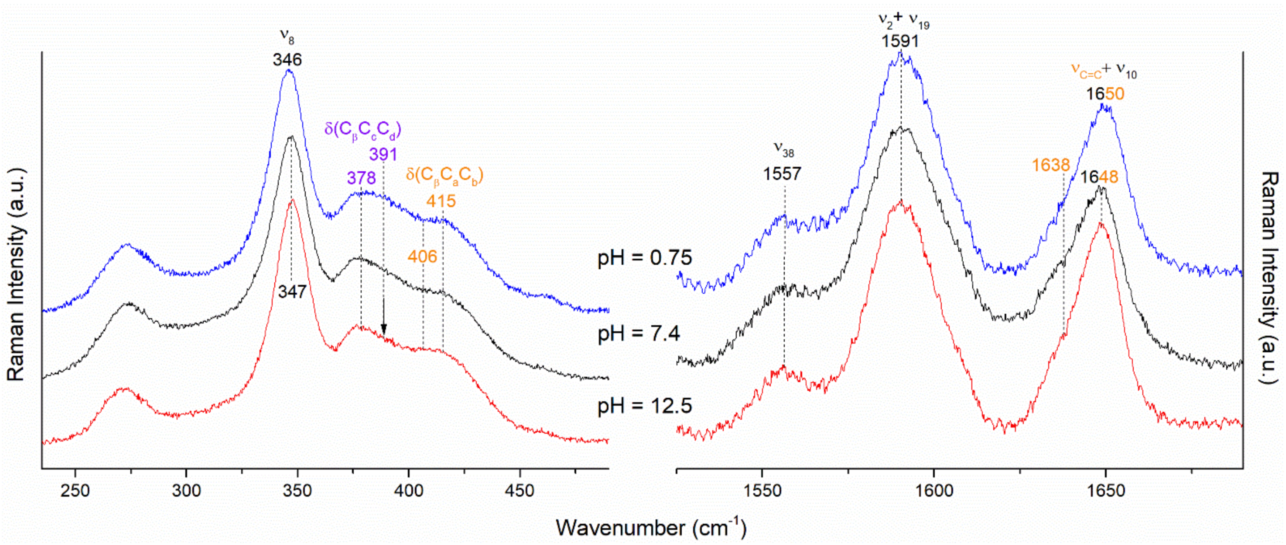


**Figure S15.** Comparison of the RR spectra of Co(III)-WT at different pH values in 50 mM phosphate buffer plus 0.1 M NaCl. Spectra of Co(III)-C46AC55A are very similar (data not shown).

**Table S1:** Wavelengths (λ, nm) of the relevant spectral bands in the UV-Vis spectra, the second derivative UV-Vis spectra and MCD spectra of Co(III)-WT and Co(III)-C46AC55A at selected pH values.

| | UV-vis | | | 2nd der | MCD | | | | | | | |
|---|---|---|---|---|---|---|---|---|---|---|---|---|
| | Soret | β | α | Soret | Soret max | Soret min | Soret zerocross | β max | β min | α max | α min | α zerocross |
| Co(III)-WT | | | | | | | | | | | | |
| pH 1.0 | 430 | 540 | 569 | 431 | 419 | 443 | 433 | 533 | 548 | 563 | 583 | 573 |
| pH 4.0 | 429 | 539 | 570 | 429 | 419 | 440 | 430 | 526 | 544 | 562 | 581 | 572 |
| pH 7.0 | 423 | 535 | 567 | 425 | 414 | 435 | 425 | 522 | 541 | 559 | 578 | 568 |
| pH 10.0 | 423 | 536 | 567 | 425 | 414 | 435 | 425 | 522 | 541 | 559 | 579 | 568 |
| pH 13.0 | 426 | 540 | 570 | 427 | 419 | 440 | 430 | 525 | 544 | 563 | 583 | 573 |
| Co(III)-C44A/C55A | | | | | | | | | | | | |
| pH 1.0 | 431 | 536 | 568 | 432 | 421 | 443 | 435 | 526 | 544 | 561 | 580 | 571 |
| pH 4.0 | 427 | 536 | 566 | 427 | 417 | 439 | 429 | 524 | 542 | 559 | 579 | 570 |
| pH 7.0 | 422 | 535 | 563 | 423 | 411 | 432 | 422 | 521 | 538 | 556 | 576 | 565 |
| pH 10.0 | 422 | 535 | 565 | 423 | 412 | 433 | 423 | 522 | 540 | 557 | 578 | 567 |
| pH 13.0 | 424 | 538 | 569 | 425 | 417 | 439 | 429 | 525 | 543 | 561 | 583 | 572 |
